# Evaluation of molecular mechanisms of (*Z*)-3-(pentadec-10′-enyl)-catechol (litreol) and synthetic derivatives as inhibitors of human leukotriene biosynthesis

**DOI:** 10.1016/j.redox.2025.103880

**Published:** 2025-09-30

**Authors:** Alessia Maria Cossu, Simona Pace, Ferdinando Bruno, Lucia Abbatiello, Carmen Cerchia, Emanuele Falbo, Alejandra Catalina Muñoz Ramírez, Christian Kretzer, Laura Miek, Fabiana Troisi, Jana Gerstmeier, Pasquale Ambrosino, Silvia Zappavigna, Antonio La vecchia, Oliver Werz, Michele Caraglia, Rosanna Filosa

**Affiliations:** a Laboratory of Precision and Molecular Oncology, Biogem Scarl, Institute of Genetic Research, 83031, Ariano Irpino, Italy; b Department of Precision Medicine, University of Campania “Luigi Vanvitelli”, Via L. De Crecchio, 7, 80138, Naples, Italy; c Institute of Pharmacy, Friedrich-Schiller-University Jena, Philosophenweg 14, D-7743, Jena, Germany; d Department of Science and Technology, University of Sannio, 82030, Benevento, Italy; e Department of Pharmacy, “Drug Discovery” Laboratory, University of Naples “Federico II”, Via D. Montesano 49, 80131, Napoli, Italy; f Department of Environmental Sciences, Faculty of Chemistry and Biology, University of Santiago de Chile, 8320000, Santiago, Chile; g Istituti Clinici Scientifici Maugeri IRCCS, Cardiac Rehabilitation Unit of Telese Terme Institute, 82037, Telese Terme, Italy; h Istituti Clinici Scientifici Maugeri IRCCS, Scientific Directorate of Telese Terme Institute, 82037, Telese Terme, Italy

**Keywords:** Leukotrienes, 5-Lipoxygenase, Inflammation, Litreol, Leukocytes, Chronic disease, Outcome, Natural compounds

## Abstract

5-Lipoxygenase (5-LO) catalyzes the early steps of leukotriene (LT) biosynthesis, making it an attractive target for anti-inflammatory drug development. This study provides a more detailed evaluation of the molecular mechanisms and pharmacological effects of litreol (CI), a natural compound from the Anacardiaceae family, along with its synthetic derivatives (CS, AS, and AI). The synthesis and biological evaluation of litreol analogs have already been previously published. Therefore, the aim of this article is to further explore their mechanisms of action, providing a more thorough investigation into their effects on 5-LO. Using both isolated human recombinant 5-LO in cell-free systems and cell-based assays, we evaluated the impact of the synthesized compounds on 5-LO product formation. Among them, CI and CS emerged as potent inhibitors, exhibiting IC_50_ values of 0.26 μM and 0.80 μM in neutrophils, and 0.06 μM and 0.15 μM in cell-free assays, respectively. Notably, CI exhibited 2.5- to 3-fold greater potency compared to its hydrogenated analogue, CS. Both compounds also showed inhibitory activity against 12-lipoxygenase (12-LO) with IC_50_ of 3.15 and 5.10 μM, respectively. Moreover, CI prevented the 5-LO/FLAP protein interaction and blocked both ERK-1/2 and p38 MAP kinase-dependent pathways required for 5-LO activation. Conversely, AS and AI derivatives did not show significant 5-LO inhibitory effects. Computational studies revealed that the differing binding modes and stability of CI and CS at the allosteric site of 5-LO explain their varying inhibitory effects. CI forms a stronger interaction network, supporting its higher potency, while CS shows greater flexibility and weaker interactions, correlating with lower activity. Additionally, the free catechol group is essential for activity, as its acetylation leads to loss of function. Overall, our findings highlight CI as a promising 5-LO inhibitor, in intact human leukocytes accounting for a novel potent anti-inflammatory compound.

## 1 Introduction

Inflammation is a complex process of human body activated in response to tissue damage, which is regulated by multiple signalling pathways, along with recruitment of different cell types including neutrophils, macrophages, and mast cells, through the secretion of specific pro-inflammatory mediators including eicosanoids, in particular leukotrienes (LTs) and prostaglandin (PG)E_2_. Two important enzymatic routes are involved in eicosanoid biosynthesis: the 5-lipoxygenase (5-LO) and cyclooxygenase (COX) pathway. 5-LO plays an essential role in the biosynthesis of leukotrienes. It catalyzes the transformation of arachidonic acid (AA), liberated by cytosolic phospholipase (cPLA_2_), to 5-hydroperoxyeicosatetraenoic acid (5-HPETE). 5-HPETE is converted to the epoxide LTA_4_ and then to LTB_4_ or to the cysteinyl-LTs C_4_, D_4_ and E_4_ depending on cell type and enzyme expression.

5-LO is expressed in polymorphonuclear leukocytes (PMNL), monocytes/macrophages, dendritic cells and in B-lymphocytes [1]. LTs play important roles during inflammatory and allergic reactions [2]. Indeed, the use of inhibitors against LT biosynthetic enzymes (i.e., 5-LO, LTA_4_ hydrolase, or LTB_4_ synthase) is considered useful for the treatment of chronic disabling diseases, including asthma, allergic rhinitis, arthritis, neuroinflammatory disorders, cardiovascular and cancer disease. Zileuton, a hydroxyurea derivative with a benzothiophene core, is a 5-LO inhibitor indicated for asthma treatment [3]. In addition to zileuton, several other 5-LO inhibitors have been reported in the literature. In details, licofelone is a dual COX/5-LO inhibitor investigated for inflammatory diseases and AA-861 is a direct 5-LO inhibitor derived from *Streptomyces* species while nordihydroguaiaretic acid (NDGA) is a non-selective antioxidant compound with 5-LO inhibitory properties. Other natural or synthetic compounds such as caffeic acid phenethyl ester (CAPE) and Atreleuton (ABT-761), have also demonstrated 5-LO inhibitory activity in preclinical or clinical studies. Although zileuton remains the only FDA-approved 5-LO inhibitor for clinical use, these alternative compounds continue to provide valuable insights into 5-LO modulation and its therapeutic potential in inflammatory and neoplastic diseases [4,5].

Other lipid mediators with potent anti-inflammatory properties like specialized pro-resolving mediators (SPM) are synthesized by 12-LO and 15-LO with the combined action of 5-LO during cell-cell interactions. For instance, lipoxins are SPM that function as stop signals for anti-inflammatory responses [6]. 5-LO-activating protein (FLAP) is involved in the biosynthesis of LT which is an integral membrane protein that represents a very important target for the development of new selective LT biosynthesis inhibitors [7]. FLAP is a member of the MAPEG (membrane associated proteins in eicosanoid and glutathione metabolism) family which facilitates AA substrate transfer to 5-LO forms a complex with 5-LO at sites where AA is liberated. FLAP inhibitors (e.g., MK886, BAY-X-1005) efficiently abolish LT formation and are proved to be effective in preclinical and clinical treatment of asthma [8].

In PG biosynthesis, microsomal PGE_2_ synthase (mPGES)-1, a membrane-bound enzyme, is responsible for PGE_2_ formation, and the inhibition of this enzyme is considered a better pharmacological strategy than COX 1/2 inhibition. AA is converted by cyclooxygenase (COX)-1 and −2 to PGH_2_ that serves as substrate for mPGES-1 that in turn is responsible for PGE_2_ formation [9]. LTs and PGE_2_ are the most important pro-inflammatory lipid mediators that are the major targets of pharmacological approaches for intervention with inflammatory disorders.

Several natural compounds represent the starting points for multi-target drug discovery due to their structural diversity and biological activity. These molecules could interact with multiple molecular targets modulating various signalling pathways simultaneously, making them particularly valuable in addressing complex diseases such as cancer, neurodegenerative disorders and inflammatory conditions. It has been demonstrated that many plant-derived polyphenolic natural products, such as epigallocatechin gallate, quercetin, gallic acid, curcumin, eugenol, and alkyl resorcinol, possesses anti-inflammatory properties such as blockade of signalling molecules (glycogen synthase kinase-3β, MAPK family members) and transcription factors (NF-κB, Nrf2), inhibition of cytokine release (IL-1β, TNFα), antioxidant activities and suppression of pro-inflammatory eicosanoid formation with a potent inhibitory activity against 5-LO [[10], [11], [12]].

Our research group has long been investigated the intricated mechanism of inflammation and how natural compounds represent an inexhaustible source of biologically active molecules with anti-inflammatory activity [5,13]. In this regard, starting from embelin, a natural derivative from *Embelia ribes* that inhibits both 5-LO and mPGES-1 activity (IC_50_ = 60 and 200 nM respectively) [14], we have discovered the ortho-quinone derivative RF 22c that potently inhibits LT formation in cellular and blood assays [15,16]. Further structural optimizations revealed the catechol analogue EA-110C RED [17] as a lead structure molecule for new anti-inflammatory strategies [18,19]. Building from these results, we introduced AOX-03 as one of most efficient suppressors of 5-LO product formation [20].

A structure–activity relationship analysis revealed two key factors critical for 5-LO inhibition: the catechol group, which appears to influence potency and the alkyl chain length and structure [17]. (Chart.1 in S.I.)

Based on this rationale, we investigated the profile of litreol ((Z)-3-(pentadec-10′-enyl)-catechol, CI), a compound isolated from *Lithraea caustica*, a member of the Anacardiaceae family known to contain several biologically active phenolic lipids, including alkylcatechols and alkylresorcinols [[21], [22], [23], [24]].

Litreol was obtained from the epicuticular extract of leaves, and three hemisynthetic derivatives were prepared: CS (3-n-pentadecylcatechol), AI ((Z)-1,2-diacetoxy-3-(pentadec-10′-enyl)-benzene), and AS (1,2-diacetoxy-3-pentadecylbenzene).

**Fig. 1:**
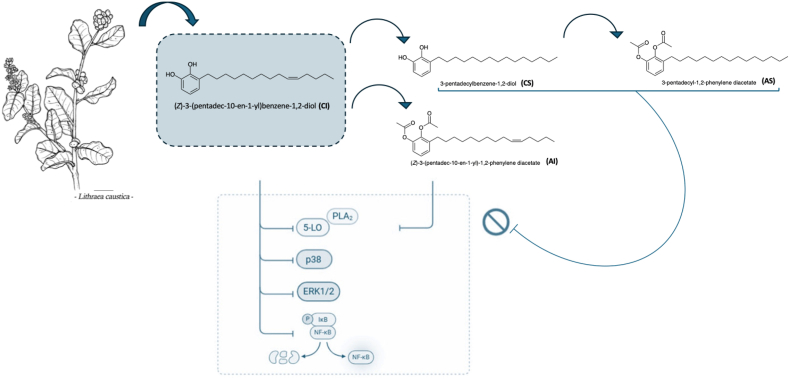
Structure-activity relationship analysis of litreol isolated from *Lithraea caustica*, and its synthetic derivatives in suppressing leukotriene biosynthesis and associated inflammatory pathways.

The inhibitory activities of these four compounds against 5-LO were determined, with litreol (IC_50_ = 2.09 μM) and CS (IC_50_ = 2.74 μM) showing the most promising results [25]. (Fig. 1) Encouraged by these findings, we resynthesized and full characterized the four compounds (CI, CS, AI, AS) and evaluated their effects in various cell-free and cell-based inflammatory assays. This study provides a comprehensive characterization of the pharmacological properties and molecular mechanisms of action of litreol and its synthetic derivatives as potent and selective 5-LO inhibitors, aiming to elucidate their role in suppressing leukotriene biosynthesis and associated inflammatory pathways.

## 2 Materials and methods

### 2.1 Plant material

Representative samples of leaves of *Lithraea caustica* (Mol) Hooker & Arnott, were collected during the flowering season, from a population growing in Farellones, Metropolitan region, Santiago, Chile (33° 18′ 35.9″S; 70° 19′ 19.9″W) at altitudes of 1200–1300 m above sea level. Voucher specimens was deposited in the Herbarium of the National Natural History Museum, Santiago, Chile.

### 2.2 Extraction of leaves

Cuticular components of L. caustica were extracted by dipping fresh plant material (1.37 kg) in cold CH_2_Cl_2_ for 5 min. After filtration, the extract was dried over anhydrous Na_2_SO_4_, and the solvent was removed under reduced pressure to yield a cuticular residue. A portion (6.1 g) was purified by flash chromatography on silica gel (20 g, 230–400 mesh) using a gradient of PE, PE–CH_2_Cl_2_ (90:10 to 10:90), CH_2_Cl_2_, and CH_3_OH–CH_2_Cl_2_ (5:95 to 10:90), affording 77 fractions. Phenolic-containing fractions (FeCl_3_ 5 % positive on TLC) were pooled (190 mg). GC-MS analysis revealed one major compound (90 %) with a molecular ion at *m/z* 318 (C_21_H_34_O_2_) and a base peak at *m/z* 123 (C_7_H_7_O_2_).

^1^H NMR and COSY spectra of the pure compound confirmed the identity as 3-(pentadec-10′-enyl)-catechol (CI), previously reported from L. caustica stem bark [26].

### 2.3 Chemistry

All reagents were of analytical grade and purchased from Sigma-Aldrich (Milano-Italy). Flash chromatography was performed on Carlo Erba silica gel 20g (230–400 mesh: CarloErba, Milan, Italy). TLC was carried out using plates coated with silica gel 60 F_254_ nm purchased from Merck (Darmstadt, Germany). Reaction yields refer to chromatographically and spectroscopically pure products. ^1^H and ^13^C NMR spectra were registered respectively on a Bruker AC 300 and 400 Hz. Chemical shifts are reported in ppm relative to tetra-methylsilane. The abbreviations used are as follows: s, singlet; d, doublet; dd double doublet; bs, broad signal.

#### 2.3.1 Synthesis of 3-n-pentadecylcatechol (CS)

**Scheme 1:**
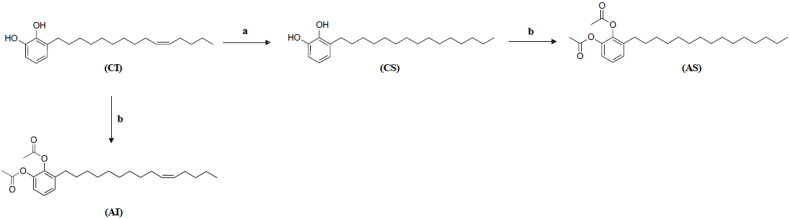
Synthesis of litreol (CI) structural analogs. Reagent conditions: (a) CH_2_Cl_2_, H_2_, Pd/C, rt; (b) Pyridine, acetyl chloride, rt.

A solution of (Z)-3-(pentadec-10-enyl)-catechol (CI) in CH_2_Cl_2_ with Pd/C was stirred under a hydrogen atmosphere at room temperature for 3 h. After filtration and concentration, the crude product was purified by flash chromatography using a hexane/ethyl acetate gradient. The appropriate fractions were combined to yield pure 3-n-pentadecylcatechol (CS) in 62 % yield; solid, mp = 60 °C (Scheme 1). ^1^H NMR (300 MHz, MeOD) δ 6.66–6.53 (m, 3H, CH), 2.57 (t, J_H-H_ = 7.7 Hz, 2H, CH_2_), 1.58 (t, J_H-H_ = 7.7 Hz, 2H, CH_2_), 1.28 (m, 22H, CH_2_), 0.88 (t, J_H-H_ = 6.72 Hz, 3H, CH_3._^13^C NMR (300 MHz, MeOD) δ: 145,82 (s, C); 144,19 (s, C); 130,62 (s, C); 121,87 (s, CH); 120,08 (s, CH); 113,57 (s, CH); 33,06 (s, CH_2_); 31,09–30,46 (m, 12C, CH_2_); 23,72 (s, CH_2_); 14,47 (s, CH_3_).

#### 2.3.2 Synthesis of 1,2- diacetoxy-3-pentadecylbenzene (AS)

A stirred solution of (CS) in dry pyridine was cooled to 0 °C under a nitrogen atmosphere. Acetyl chloride was added dropwise via syringe, and the mixture was stirred for 4 h at room temperature. The reaction was diluted with ethyl acetate and washed successively with water, 1 N HCl, saturated aqueous NaHCO_3_, and brine. The organic layer was dried over anhydrous Na_2_SO_4_, filtered, and concentrated under reduced pressure. The crude product was purified by flash chromatography using a hexane/ethyl acetate gradient. The appropriate fractions were combined and concentrated to yield 1,2-diacetyl-3-pentadecylbenzene (AS) in 90 % yield, solid, mp = 45 °C (Scheme 1). ^1^H NMR (300 MHz, MeOD) δ 7.25–7.09 (m, 2H, CH), 7.03 (d, J_H-H_ = 7.7 Hz, 1H, CH), 2.53 (t, J_H-H_ = 7.8 Hz, 2H, CH_2_), 2.29 (s, 1H, CH), 2.24 (s, 1H, CH), 1.55 (t, J_H-H_ = 7.1 Hz, 2H, CH_2_), 1.28 (m, 22H, CH_2_), 0.89 (t, J_H-H_ = 6.7 Hz, 3H, CH_3_^13^C NMR (300 MHz, MeOD) δ: 169,96 (s, C=O); 169,91 (s, C=O); 143,97 (s, C); 142,01 (s, C); 137,88 (s, C); 128,29 (s, CH); 127,11 (s, CH); 122,04 (s, CH); 33,02 (s, CH_2_); 31,08–30,32 (m, 12C, CH_2_); 23,68 (s, CH_2_); 20,48 (s, CH_3_); 20,19 (s, CH_3_); 14,39 (s, CH_3_).

#### 2.3.3 Synthesis of (*Z*)-1,2-diacetoxy-3-(pentadec-10′-enyl)-benzene (AI)

A stirred solution of (CI) in dry pyridine was cooled to 0 °C under a nitrogen atmosphere. Acetyl chloride was added dropwise via syringe, and the mixture was stirred for 4 h at room temperature. The reaction was diluted with ethyl acetate and washed successively with water, 1 N HCl, saturated aqueous NaHCO_3_, and brine. The organic layer was dried over anhydrous Na_2_SO_4_, filtered, and concentrated under reduced pressure. The crude product was purified by flash chromatography using a hexane/ethyl acetate gradient, and the appropriate fractions were combined and concentrated to give (*Z*)-1,2-diacetyl- 3-(pentadec-10’ -enyl)-benzene (AI), in 80 %, oil (Scheme 1).

^1^H NMR (300 MHz, MeOD) δ 7.25–7.10 (m, 2H, CH), 7.03 (d, *J* = 9.0 Hz, 1H, CH), 5.34 (m, 2H, CH), 2.53 (t, *J* = 7.9 Hz, 2H, CH_2_), 2.29 (s, 3H, CH_3_), 2.24 (s, 3H, CH_3_), 2.06–1.99 (m, 4H, CH_2_), 1.55 (m, 2H, CH_2_), 1.38–1.25 (m, 16H, CH_2_), 0.90 (t, *J* = 7.1 Hz, 3H, CH_3_). ^13^C NMR (300 MHz, MeOD) δ: 169,97 (s, C=O); 169,91 (s, C=O); 143,97 (s, C); 142,01 (s, C); 137,88 (s, C); 130,76 (s, CH); 130,73 (s, CH); 128,29 (s, CH); 127,11 (s, CH); 122,04 (s, CH); 33,07 (s, CH_2_); 31,07–30,23 (m, 8C, CH_2_); 28,03 (s, CH_2_); 27,81 (s, CH_2_); 23,29 (s, CH_2_); 20,47 (s, CH_3_); 20,19 (s, CH_3_); 14,27 (s, CH_3_).

Further details regarding the mass spectra, as well as the ^1^H, ^13^C, and COSY NMR spectra, are provided in the Supporting Information.

### 2.4 Cells

PMNL and monocytes were isolated from human peripheral blood of adult healthy volunteers, with consent, obtained from the Institute of Transfusion Medi-cine, University Hospital Jena (Germany). PMNL were immediately isolated by dextran sedi-mentation, centrifugation on Nycoprep cushions, and hypotonic lysis of erythrocytes, as described [27]. PMNL viability was analyzed by trypan blue exclusion with a Vi-cell counter (Beckmann Coulter GmbH, Krefeld). HEK-293 cells were grown in DMEM/High Glucose (4.5 g/L) medium supplemented with heat-inactivated fetal calf serum (FCS, 10 %, vv_1), 100 U/mLpenicillin, and 100 lg/mL streptomycin at 37 °C in a 5 % CO_2_ incubator. HEK-293 cells were stably transfected with 5-LO and FLAP as validated experimental cell-based model for studying the regulation of 5-LO [28]. None of the cell lines used in this study were authenticated.

### 2.5 Isolation of human immune cells and cell-based assay for 5-LO activity determination

Freshly isolated human PMNL (5 × 10^6^) were resuspended in 1 mL PBS buffer containing 1 mg/mL glucose (PG buffer). After pre-incubation with litreol compounds (15 min, 37 °C), 5-LO product formation was started by addition of 1 mM CaCl_2_ and 2.5 μM A23187 with or without AA (20 μM). After 10 min at 37 °C, the reaction was stopped with 1 mL of methanol for 10 min on ice and LO products formed and released in the supernatants were extracted and analyzed by HPLC. In detail, after centrifugation (800g, 10 min, room temperature) the samples were applied to C-18 solid-phase extraction columns (100 mg; IST, Mid Glamorgan, United Kingdom), preconditioned with 1 mL methanol and 1 mL water. The columns were washed with 1 mL water and 1 mL water/methanol (75/25, vol/vol) and 5-LO metabolites were eluted with 300 μL methanol. The extract was then diluted with 120 μL water, and 100 μL diluted extract were analyzed by HPLC using a C-18 Radial-Pak column (Waters) eluted with methanol/water/acetic acid 75/25/0.1 (vol/vol/vol) at a flow rate of 1.2 mL/min. Amounts of different metabolites were determined by peak area integration. 5-LO products analyzed include LTB_4_ and its two all-trans isomers, 5(S)-hydroxy-6-*trans*-8,11,14-cis-eicosatetraenoic acid (5-HETE), and 5(S)-hydroper-oxy-6-trans-8,11,14-*cis*-eicosatetraenoic acid (5-HpETE). 5-HETE and 5-HpETE coelute as one major peak, and the integration of this peak represented both eicosanoids.

### 2.6 Expression, purification, and activity assay of human recombinant 5-LO

**5-LO was expressed in E. coli B121 (DE3) transformed with pT3- 5LO plasmid, and purification of 5-LO was performed as described previously.** [29] **Briefly, E. coli was lysed in 50 mM tri-ethanolamine/HCl,** pH **8.0, 5 mM EDTA, 60 mg/mL soybean trypsin inhibitor, 1 mM phenylmethanesulfonyl fluoride, 1 mM dithiothreitol and 1 mg/mL lysozyme and then sonified (3 × 15 s). The homogenate was then centrifuged at 40,000g for 20 min at 4°C. 5-LO in the supernatant was partially purified by affinity chromatography on an ATP-agarose column (Sigma-Aldrich). Semi-purified 5-LO was diluted in PBS containing EDTA (1 mM) and ATP (1 mM) and immediately used for 5-LO activity assays. Samples were pre-incubated with the test compounds or vehicle (0.1 % DMSO) for 10 min at 4°C. Samples were pre-warmed for 30 s at 37°C, and 2 mM CaCl_2_ plus the indicated concentrations of AA were added to start 5-LO product formation. The reaction was stopped after 10 min by addition of one volume of ice-cold methanol, and the formed metabolites were analyzed by HPLC. 5- LO products include the all-trans isomers of LTB4 as well as 5-HPETE and its corresponding alcohol 5-HETE**.

### 2.7 Lactate dehydrogenase release (LDH assay)

After treatment of PMNL, supernatants were centrifuged (600g × 5 min, 4 °C), and 100 μL of supernatant were mixed with 200 μL LDH reaction buffer (75 mM Tris/HCl, pH 7.4, 0.3 mM NADH, 1.5 mM Na-pyruvate) in a 96-well plate. The decrease of NADH was measured immediately by absorbance at 340 nm in a kinetic mode (every 7 s for 20 min; Multiskan Spectrum). As a positive control for maximal LDH release, cells treated with 1 % Triton X-100 were included in each experiment, while untreated cells and vehicle (DMSO) served as negative controls.

### 2.8 Determination of the formation of reactive oxygen species (ROS) in neutrophils

Neutrophils (10^7^ cells/mL in PG buffer) were pre-incubated for 15 min at 37 °C with litreol compounds, diphenyleneiodonium chloride (DPI, used as a positive control for ROS inhibition), or 0.1 % DMSO as vehicle control. Then, 2′,7′-dichlorofluorescence-diacetate (DCF-DA; 1 μg/mL) and CaCl_2_ (1 mM) were added 2 min prior to addition of 0.1 μM phorbol myristate acetate (PMA, AppliChem, Darmstadt, Germany). The fluorescence emission at 530 nm was measured after excitation at 485 nm in a thermally controlled (37 °C) NOVOstar microplate reader (BMG Labtechnologies GmbH, Offenburg, Germany).

### 2.9 Western blot analysis

Human polymorphonuclear leukocytes (PMNLs; 50 × 10^6^), isolated from whole blood, were resuspended in 1 mL of cold PGC buffer. Briefly, neutrophils were pre-incubated for 15 min at 37 °C with test compounds (1 μM). PMNL were stimulated with fMLP (1 μM) for 90 s and chilled on ice. The samples were subsequent centrifuged (1200g, 10 min) and the pellets were resuspended in 100 μL od SDS-PAGE loading buffer (2x) (Tris 1 M pH 8, EDTA 0.2 M, 10 % SDS, 10 % mercaptoethanol). Correct protein loading on the gels and transfer of proteins to the nitrocellulose membrane were confirmed by Ponceau staining. Membranes with transferred proteins were incubated overnight with primary antibodies in 1x Tris Buffered Saline (TBS) with 1 % Casein (Biorad- #1610782). Membranes were then washed and incubated with horseradish peroxidase-conjugated secondary antibodies for 1 h at room temperature. Blots were developed using enhanced chemiluminescence detection reagents ECL (Immobilion ECL Ultra Western HRP Substrate - Cat# WBULS0500 Millipore) and acquired using ChemiDoc Imaging System (BioRad). Antibody recognizing phosphorylated p38-MAPkinase, pERK, ERK, NfKB were from Cell Signalling Technology (Boston, MA) and used at 1:1000 dilution. Secondary antibodies were purchased from Biorad (170–6515; 170–6516) and used at 1:2000 diluition.

### 2.10 Intracellular Ca^2+^ measurements

PMNL (10^7^ x ml PG buffer) were pre-stained with Fura-2/AM (2 μM) for 45 min at 37 °C in the dark. After two washing steps, cells were resuspended in PG buffer containing 0.1 % BSA at a density of 5 × 10^6^/mL. 100 μL of the cell suspension was transferred into a 96-well plate and pre-incubated for 10 min at 37 °C with compound or vehicle (1 % DMSO), and 2 min prior stimulation 1 mM CaCl_2_ was added, following addition of 0.1 μM fMLP or 2 μM ionomycin as a positive control for Ca^2+^ influx. The signal was monitored in a thermally (37 °C) controlled NOVOstar microplate reader (BMG Labtechnologies GmbH, Offenburg, Germany) (emission at 510 nm, excitation at 340 nm (Ca^2+^-bound Fura-2) and 380 nm (free Fura-2). After cell lysis with Triton X-100 the maximal fluorescence signals were monitored and after chelating Ca^2+^ with 10 mM EDTA the minimal fluorescence signals. The concentration of Ca^2+^ was calculated from the ratio of the signals at 340 and 380 nm according to Grynkiewicz G et *al*. [30].

### 2.11 Determination of 5-LO product formation in transfected HEK293 cells

HEK-293 cells stably transfected with 5-LO and FLAP [31] were collected by trypsinization and centrifugation (1200 rpm, 10 min, 4 °C). 5-LO product synthesis was determined as follows. Cells (1 × 10^6^) were resuspended in PG buffer, preincubated with compounds or vehicle (0.1 % DMSO) for 15 min at 37 °C followed by stimulation with 2.5 μM A23187 plus 3 μM exogenous AA. The reaction was stopped by addition of 1 mL ice-cold methanol. Upon the addition of acidified PBS plus internal standard (200 ng prostaglandin B_1_), 5-LO products (all-trans isomers of LTB_4_ and 5-H(p)ETE) were extracted using C18 columns (100 mg; United Chemical Technologies, Bristol, PA, USA) and analyzed by reverse-phase HPLC using a C-18 Radial-PAK column (Waters, Eschborn, Germany) as described above for PMNL.

### 2.12 Analysis of subcellular localization of 5-LO by immunofluorescence microscopy

Subcellular localization of 5-LO and FLAP was analyzed by immunofluorescence (IF) microscopy. In brief, HEK-293 cells expressing 5-LO/FLAP were seeded onto acid-washed (50 % sulfuric acid) and poly-l-lysine (0.01 %)-coated glass coverslips and cultured for 48 h at 37 °C until ∼60 % confluency. Cells were pre-incubated with 10 μM of compounds or vehicle (0.1 % DMSO) for 10 min before stimulation with 2.5 μM A23187 for 20 min at 37 °C. After fixation with 4 % paraformaldehyde solution, followed by permeabilization by using 50 mM ammonium chloride and acetone (3 min, 4 °C), cells were incubated with 10 % non-immune goat serum blocking solution for 1 h. Then, cells were incubated over night with mouse monoclonal anti-5-LO antibody (1:100) and rabbit polyclonal anti-FLAP antibody (5 mg/mL). The coverslips were intensively washed with PBS before staining with the fluorophore-labeled secondary antibodies Alexa Fluor 488 goat anti-rabbit (1:500) and Alexa Fluor 555 goat anti-mouse (1:500) for 20 min in the dark. The coverslips were mounted on glass slides with Mowiol containing 2.5 % n-propyl gallate and 4,6-diamidin-2-phenylindol (DAPI; Invitrogen, Carlsbad, CA), a staining for nuclear DNA. Samples were analyzed by a Zeiss Axio-vert 200 M microscope, and a Plan Neofluar _ 100/1.30 Oil (DIC III) objective (Carl Zeiss, Jena, Germany). An AxioCam MR camera (Carl Zeiss) was used for image acquisition.

### 2.13 Determination of PGE_2_ in monocyte

Monocytes (1 × 10^6^ ml ‘‘monocyte medium” composed of RPMI 1640 containing penicillin (100 U/mL), streptomycin (100 μg/m/L), 2 mM l-glutamine, and 2 % human serum) were seeded in 12-well plates. After 1.5 h, 37 °C, 5 % CO_2_ cells were stimulated with 1 μg/mL LPS for 20 h, 37 °C, 6 % CO_2_. Cells were harvested and resuspended in PGC buffer, and inhibitors or vehicle (0.1 % DMSO) were added for 15 min at 37 °C. Then, cells were stimulated with 1 μM AA for 30 min. The supernatant was collected by centrifugation and analyzed for PGE_2_ by EIAs that were conducted according to the manufacturer's instructions (PGE_2_: Biotrend Chemikalien GmbH, Cologne, Germany).

### 2.14 Statistical analysis

All data are expressed as mean +S.E.M. Statistical analysis was carried out using GraphPad Prism 9 software (San Diego, CA). Paired *t*-test was used to analyze experiments for comparison of two groups; while one-way ANOVA or multiple paired t-tests were applied for multiple comparisons as indicated. A p-value ≤0.05 is a criterion for statistical significance.

### 2.15 Protein and ligand preparation

The crystal structure of stable 5-LO bound to the allosteric inhibitor 3-acetyl-11-keto-beta-boswellic acid (AKBA)(PDB6NCF) [32] was employed for docking studies. The protein structure was prepared using the Protein Preparation Wizard in Maestro (Protein Preparation Wizard; Epik, Schrödinger, LLC, New York, NY, 2023; Impact, Schrödinger, LLC, New York, NY; Prime, Schrödinger, LLC, New York, NY, 2023). Solvent molecules and other non-relevant chemical entities were removed, hydrogen atoms were added, and bond orders, charges, and atom types were corrected. The hydrogen-bonding network was refined through comprehensive sampling of rotamers, tautomers, and protonation states of titratable residues at neutral pH. A restrained minimization of hydrogen atoms was carried out on the protein structures using the Impref module with the OPLS4 force field, applying a 0.3 Å RMSD threshold from the original coordinates as a constraint. The synthesized compounds were initially drawn using the Maestro 2D-sketcher and subsequently processed with LigPrep (LigPrep, Schrödinger, LLC, New York, NY, 2023) to generate 3D conformations and relevant tautomeric states at pH 7.0. These 3D structures were then subjected to energy minimization using the OPLS4 force field.

### 2.16 Docking studies

Docking of the compounds under study was carried out with Glide module (Glide, Schrödinger, LLC, New York, NY, 2023) [33,34]. The docking grid was generated by considering a box of 10 Å × 10 Å × 10 Å surrounding the bound ligand AKBA. Docking was performed in Standard Precision (SP) mode, generating ten binding poses per compound. The final binding poses were selected based on the scoring, provided by GlideScore function, and the consistency of protein-ligand interactions with the experimental data.

Hydrogen bonds were considered when the donor–acceptor distance was ≤3.5 Å (optimal ∼2.8 Å) and the donor–hydrogen–acceptor angle was in the 150–180° range. Hydrophobic interactions were defined as non-polar contacts within 5.0 Å, with optimal distances typically between 3.5 and 4.0 Å.

Figure preparation was carried out with PyMOL (The PyMOL Molecular Graphics System, Version 2.0 Schrödinger, LLC).

### 2.17 Molecular dynamics simulations

Molecular dynamics (MD) simulations were carried out on the selected poses obtained from docking, employing explicit solvent and periodic boundary conditions. The protein utilized the ff14SB AMBER force field, and the force field parameters for the non-standard ligands within the iron active site were processed as reported by Torras et al. [35] The AMBER 2018 software was employed for all classical simulations. Initially, the system was equilibrated by keeping protein restrained to initial position using harmonic restraints with a constant force of 20 kcal/mol. Each system was subjected to minimization steps, and then subsequently heated to 298 K for 250 ps at in NVT ensemble. The numerical integration step was set to 2.0 fs, and the Langevin thermostat was used with a 5.0 ps−1 collision frequency. The system was allowed to relax for an additional 500 ps using an NPT ensemble at 298 K and 1 atm of pressure. In all cases, the bond lengths involving hydrogen atoms were kept at their equilibrium distance by means of the SHAKE algorithm. Distance cutoffs were applied at 10.0 Å. to compute the van der Waals interactions. Long-range electrostatics were computed with the particle mesh Ewald method. The constraints on the solute atoms were removed, and another trajectory was generated with constant density following the same conditions as before for a duration of 5 ns. Subsequently, production runs were conducted using an NVT ensemble at 298 K for 300 ns. The coordinates of the production trajectories were saved every 20 ps for further analysis.

### 2.18 Clustering and trajectory analysis

To preserve the meaningful information extracted from MD simulations we focused on appropriate feature spaces. In our study, the key features included distances between ligand and residues within the active site, as well as their RMSD values, which collectively constituted our feature matrix. The reference structure for computing RMSD was defined as the initial frame of each simulation. In addition to the specific bond distances between ligand and receptor, we incorporated RMSD values for backbone atoms of the protein, the ligand itself, and the interacting residues within the LBD as geometric features. To mitigate potential biases due to varying data distributions, each feature was standardized to have a mean of zero and a standard deviation of one [36]. Sampling was conducted at intervals of 10 ps to minimize short-term correlations in clustering [37,38]. Dimensionality reduction was performed using Principal Component Analysis (PCA), retaining components that explained 90 % of the total variance. Subsequently, a clustering analysis identified essential components within the feature space, identifying representative frames termed cluster centroids. The K-means++ algorithm [39] was employed throughout this analysis. Its performance was evaluated across multiple runs with values of k ranging from 2 to 10, using internal validation criteria such as Silhouette Score (SI), Dunn Index (DI), Calinski-Harabaszscore (pSF) and Within Sum of Squares error (WSS). SI, DI, and pSF should have a maximum corresponding to the parameter set (the value of k in this case) that yields the best clustering, while WSS searches for a change in slope. Through iterative evaluation, the best value of k was determined based on convergence across three out of four criteria, ensuring robust clustering performance.

## 3 Results

### 3.1 Effect of litreol compounds on the inhibition of 5-LO product biosynthesis in isolated leukocytes

**Fig. 2:**
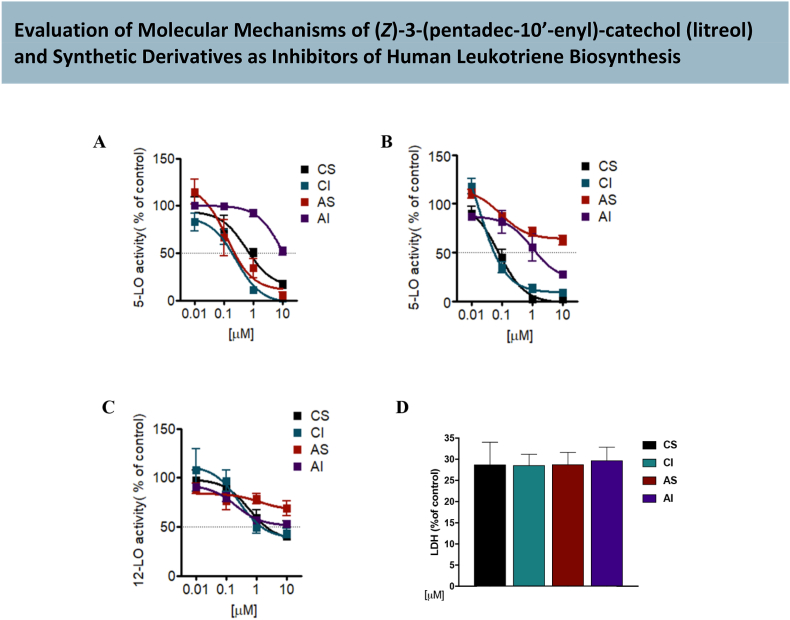
Effect of compounds on the synthesis of LO products in intact human neutrophils. Cells were preincubated with 1, 2, 3 and 4, zileuton or 0.1 % DMSO as vehicle for 15 min at 37 °C prior stimulation as indicated (a) Neutrophils (5 x 106, each) were stimulated with 2.5 μM A23187 for 10 min and 5-LO products were analyzed by HPLC (b) Neutrophils (5 x 106) were stimulated with 2.5 μM A23187 plus 20 μM AA for 10 min and 5-LO products were analyzed by HPLC. (c) Neutrophils (5 x106) were stimulated with 2.5 μM A23187 plus 20 μM AA for 10 min and 12-LO products were analyzed by HPLC. (d) Effect of 1, 2, 3 and 4 on neutrophils cytotoxicity. Data are expressed as percentage of control (100 %), means ± S.E.M., n = 3–5. ∗p < 0.05; ∗∗∗p < 0.001 versus vehicle control**.**

PMNL have high capacities to produce 5-LO products, including leukotrienes, which play roles in the regulation of inflammatory and immune response. Due to their robust 5-LO enzymatic activity and physiological relevance in inflammation, PMNL are widely used as ex vivo cell model to study LT biosynthesis and to screen for potential inhibitors of this pathway. We assessed the inhibitory capacity of litreol (CI) and its synthetic derivatives (CS, AS and AI) to inhibit 5-LO activity in isolated human PMNL. By using PMNL we aimed to closely mimic the in vivo environment where these compounds could exert their effect, providing insights on their therapeutic potential to modulate leukotriene-mediated inflammatory processes. Our results show that CI and CS inhibited 5-LO product formation in intact PMNL stimulated by A23187 (Fig. 2A) with IC_50_ of 0.26 and 0.80 μM respectively. AS had similar activity (IC_50_ of 0.5) while AI was less active with IC_50_ > 10 μM. The addition of exogenous substrate AA (20 μM) to circumvent endogenous cPLA2-mediated AA supply for 5-LO product formation did not affect or increased the potency of the compounds CI and CS with IC_50_ of 0.09 and 0.07 μM, while AS and AI were less efficient with IC_50_ > 10 and 1.5 μM, respectively (Fig. 2B). Furthermore, as in such PMNL incubations also 12-LO products are formed, we analyzed the effect of the compounds on 12-LO activity. CI and CS inhibited also 12-LO activity with IC_50_ of 3.15 and 5.10 μM, respectively, while AS and AI had no significant effect at 10 μM (Fig. 2C; Table 1).

**Table 1:** Effects of litreol and synthetic derivatives on 5-LO activity. Data are expressed as means ± S.E. of single determinations obtained in three to four independent experiments.

Cpd	Cell-based A23187	Cell-based A23187 + AA	LTC_4_	Cell-free
**CI**	0.26 ± 0.9	0.09 ± 0.4	3.15 ± 0.8	0.06 ± 0.04
**CS**	0.80 ± 0.5	0.07 ± 0.9	5.10 ± 1.3	0.15 ± 0.5
**AI**	>10	1.5 ± 1.4	>10	>10
**AS**	0.5 ± 0.5	>10	>10	>10
**Zileuton**	0.9 ± 0.3	2.5 ± 1.2	–	0.59 ± 0.10

LDH release assays confirmed that none of the assessed compounds, CI and its synthetic derivatives CS, AS, and AI, induced cytotoxic effects on human PMNLs. LDH levels in the supernatants remained comparable to those of untreated controls, indicating preserved cell membrane integrity and the absence of lytic cell death (Fig. 2D).

### 3.2 Effect of litreol compounds on 5-LO inhibition in cell-free test system

**Fig. 3:**
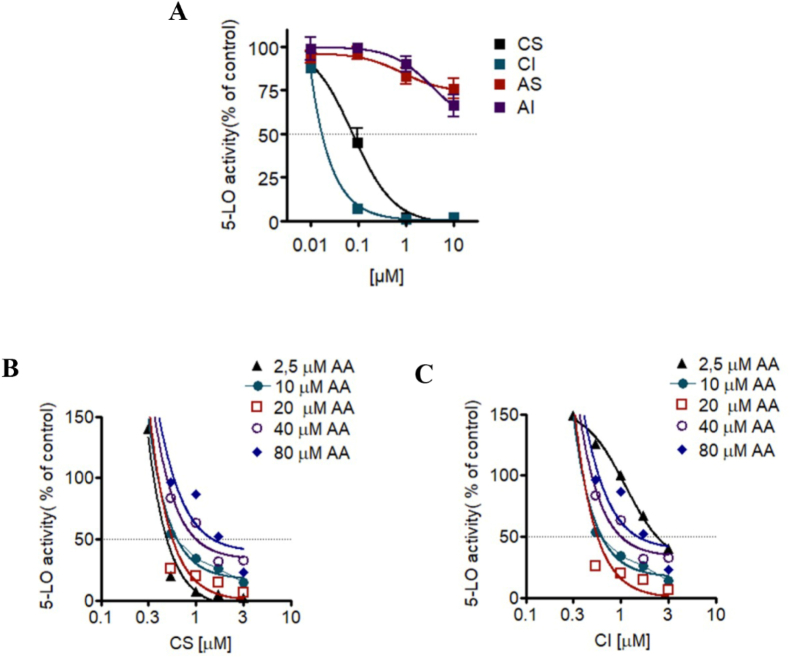
(A) Effect of compounds on 5-LO product synthesis in cell-free assay. Purified 5-LO was incubated with 1, 2, 3 and 4 or vehicle (DMSO 0,1 %) at 4 °C for 15 min. Samples were pre-warmed for 30s at 37 °C, 2 mM CaCl2 and 20 μM AA were added, and after 10 min 5-LO product formation was determined. Data are expressed as percentage of control (100 %) mean ± S.E.M., n = 3–4. Effect of 1 and 2 on 5-LO product synthesis in cell-free assay. Purified 5-LO was incubated with CS **(B)** and CI **(C)** or vehicle (DMSO 0,1 %) for 15 min at 4 °C and stimulated with varying concentrations of AA. Data are expressed as percentage of control (100 %) mean ± S.E.M., n = 3–4.

Since the compounds showed prominent inhibition of 5-lipoxygenase (5-LO) product formation in PMNL, we also evaluated their efficacy in a cell-free 5-LO activity assay using purified human recombinant 5-LO enzyme. This approach allowed us to determine whether the inhibition observed in PMNL was due to a direct interaction with the 5-LO enzyme or mediated by other cellular factors or metabolic pathways. Moreover, the cell-free assay enabled the assessment of the compounds direct effect on the catalytic activity of the enzyme, eliminating potential interference from indirect mechanisms or effects on other cellular components. Under cell-free conditions CI and CS repressed 5-LO activity, with IC_50_ of 0.06 and 0.16 μM, respectively. These findings suggest that CS and CI are more effective in a cell-free environment. No significant 5-LO inhibition was found for AS and AI (up to 10 μM) in the cell free assay (Fig. 3A). Then, we explored the mechanism of inhibition of 5-LO activity in more detail. To investigate a possible competition with the substratefor 5-LO we increased the concentrations of AA (2.5, 5, 10, 20, 40 and 80 μM) in assays using isolated 5-LO. Our results showed that the maximal inhibitory potency of CI (Fig. 3C) was reached at 20 μM AA, excluding a substrate competitive mechanism with 5-LO. Similarly, the efficiency of derivative CS to inhibit 5-LO in the cell-free assay was not competitive although the highest potency was reached at 2.5 μM AA as a substrate (Fig. 3B).

### 3.3 Effects of litreol compounds on ROS formation

**Fig. 4:**
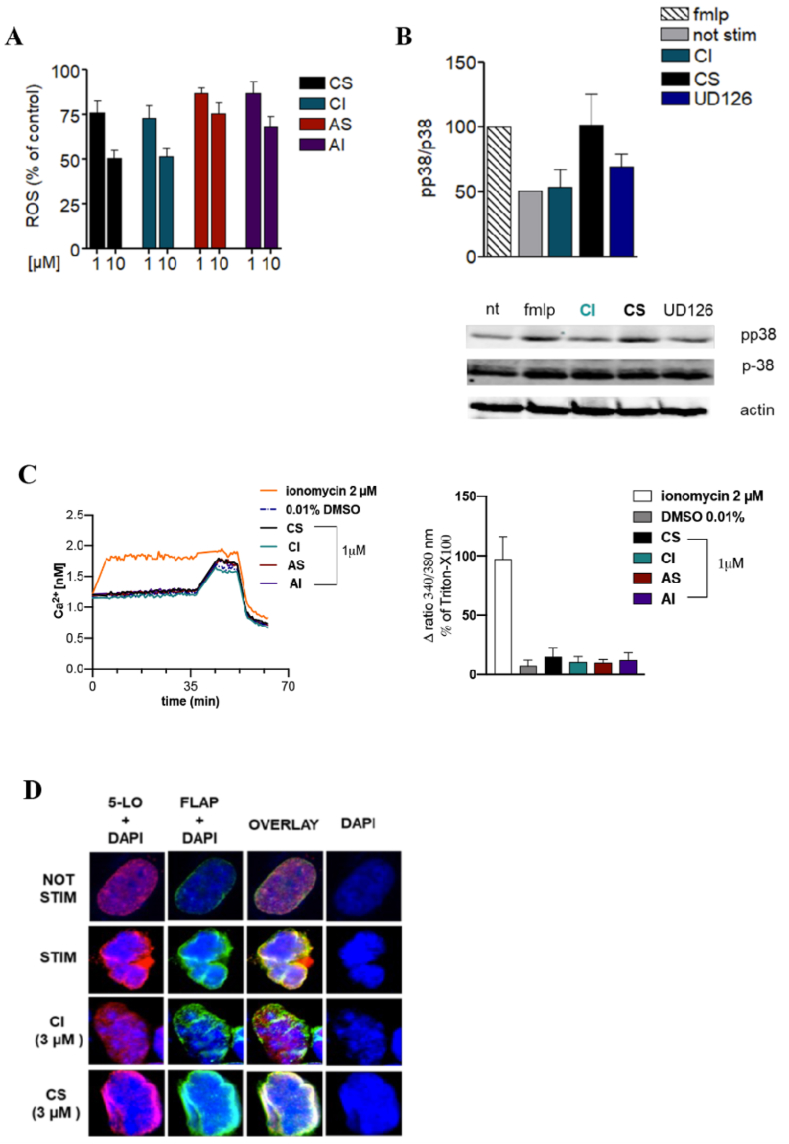
(**A**) Effect of CS, CI, AS, and AI on ROS formation. Neutrophils were pre-incubated with CS, CI, AS and AI (or 0,1 % DMSO as vehicle control) for 15 min, loaded with the fluorescent dye DCF-DA and stimulated with 0,1 μM PMA. The relative increase in fluorescence was determined after 360s at 37 °C. Data means S.E.M., n = 3. (**B**) Effect of CI and CS on 5-LO subcellular distribution, p-38 activation and Ca2+ mobilization. Neutrophils were preincubated with 1 μM CI and CS or 0.1 % DMSO for 15 min at 37 °C followed by stimulation with 1 μM fMLP for 15 min. The amounts of phosphor-p38 and p38 (for normalization) were analyzed by Western Blot. Data obtain by densitometry (bar charts, mean +S.E.M.; n = 3) are expressed percentage of fMLP-stimulated control. (C) Fura- 2/AM-loaded human neutrophils in PBS containing 1 mM Ca^2+^ were preincubated with 2 μM inonomycin or vehicle (0.01 % DMSO) or with 1 μM CI and CS at 37 °C and the baseline recorded for 30min. Exemplary time courses of the ratio of absorbance at 340 vs. 380 nm, reflecting [Ca^2+^], are shown. The ratio of absorbance at 340 vs. 380 nm is given as percentage of cells that were lysed with Triton X-100 (=100 % control). Data are given as means ± S.E.M., n = 3 separate donors. (**D**) Effect of CI and CS on 5-LO subcellular redistribution, 5-LO/FLAP interaction. Subcellular localization of 5-LO and FLAP was monitored by indirect IF microscopy in human HEK 293 cell pre-treated with 3 μM CI, CS or vehicle (10 min) and activated with 2.5 μM A23187 for 3 min. Images show single staining for 5-LO (red, top lane), FLAP (green, middle lane), and overlay of 5-LO and FLAP (bottom lane). Results are representative for 100 individual cells of three independent experiments: scale bar = 10 μm.

5-LO activity is highly sensitive to redox regulation and a certain peroxide threshold in the cell is crucial for full activity of the enzyme. Therefore, to better understand whether the litreol derivatives affect 5-LO activity indirectly by modulating cellular redox status, we evaluated their ability to interfere with reactive oxygen species (ROS) formation in intact PMNL, stimulated with PMA. This analysis helped to clarify if the observer 5-LO inhibition is related to antioxidant properties of compounds or to a direct enzymatic inhibition. Our results showed that all compounds (at 1 and 10 μM) failed to affect ROS formation in PMA-stimulated PMNL as compared to the DPI control (10 μM, not shown), and, in unstimulated cells, the compounds did not induce ROS formation (Fig. 4A).

### 3.4 Impact of litreol compounds to modulate 5-LO activation pathways

**Fig. 5:**
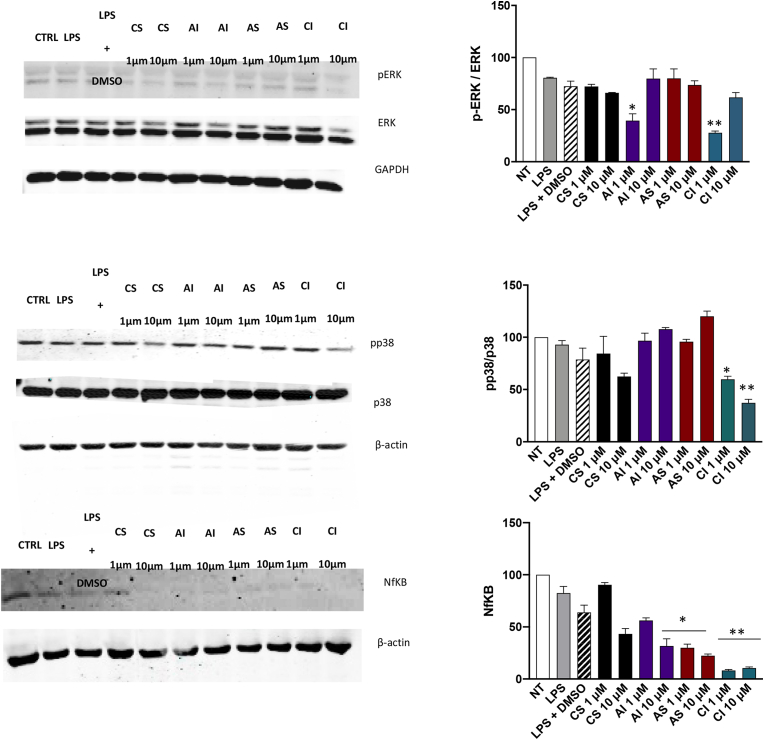
Modulation of p38-MAPK, ERK, and NF-κB by litreol compounds under pro-inflammatory stimuli. Western blot analysis of total and phosphorylated ERK (*p*-ERK), p38 MAPK, and NFκB in LPS-stimulated cells treated with different litreol derivatives. The experiments were repeated at least three times giving always similar results. Columns represent the intensity of the different bands evaluated as arbitrary units. Results are given as means ± S.E.M., n = 3.

To gain additional insights into the mechanism by which litreol compounds inhibit 5-L0 production formation, we investigated their potential effects on key intracellular signalling pathways that contribute to 5-LO modulation within the cells. Specifically, we focused on two major modulators of 5-LO activity: the p38 mitogen-activated protein kinase (MAPK) pathway and intracellular calcium (Ca^2+^) levels, both of which are essential for full enzyme activation and LT synthesis. In addition to p38 MAPK, we also investigated the activation status of other important signalling molecules involved in inflammatory responses and 5-LO regulation, including phosphorylated ERK (*p*-ERK), total ERK, and NF-κB, across all litreol derivatives under LPS stimulation. In fact, LPS is a potent activator of innate immune responses through TLR4 engagement and leads to robust activation of NF-κB and MAPK pathways. (Fig. 5). Western blot data revealed a strong inhibition of NF-κB expression by all derivatives, with the most pronounced effect observed for compound CI. Notably, CI also significantly inhibited the phosphorylation and expression of both p38 MAPK and ERK, unlike the other derivatives which did not markedly affect these kinase activity. Based on these preliminary screening results, we focused our next analyses on the two compounds CS and CI, as they have showed the most promising effects. Phosphorylation of 5-LO by p38 MAPK has been shown to enhance its catalytic activity and promote LT biosynthesis. Therefore, we addressed whether CS and CI might interfere with p38 MAPK pathway-dependent 5-LOphosphorylation induced by fMLP, a well-established stimulus for PMNL and 5-LO activation. Western blot analysis showed that CI compound inhibited fMPL-induced activation (=phosphorylation) of p38 MAP kinase and ERK in PMNL (Fig. 4B) while CS did not show significant effects. This inhibitory profile of CI suggested that it exerted a more comprehensive modulation of inflammatory signalling pathways, targeting key kinases and transcription factors involved in the regulation of 5-LO activity and LT biosynthesis. The ability of CI to suppress NF-κB, p38 MAPK, and ERK activation likely contributed to its enhanced anti-inflammatory potential compared to the other litreol derivatives, that had a more selective and/or limited effects on these pathways. Intracellular Ca^2+^ mobilization is another key step in 5-LO activation, as increased Ca^2+^ levels promote the translocation of 5-LO form the cytoplasm to the nuclear membrane, where it becomes catalytically active. To assess whether the litreol compounds influenced this pathway, we measured intracellular Ca^2+^ levels in PMNL following stimulation. Neither CS nor CI caused significant changes in Ca^2+^ concentrations (Fig. 4C), excluding the possibility that their inhibitory effects on 5-LO product formation are mediated by interference with calcium-dependent signalling.

### 3.5 Effect of litreol compounds on subcellular localization of 5-LO and FLAP

Based on the previously obtained results on the regulation of 5-LO activity and its upstream signalling cascades by litreol derivatives, we next explored their impact on the subcellular localization of 5-LO and its activating partner FLAP. Besides the absolute supply of substrate in intact cells, the accessibility of AA for 5-LO is tightly regulated by FLAP, which facilitates the transfer of AA to 5-LO at the nuclear envelope. Therefore, FLAP inhibitors prevent the formation of 5-LO/FLAP complex, effectively blocking 5-LO product formation in cells. In agreement with our previous findings on the inhibitory effects of CI on5-LO activity and signaling, immunofluorescence microscopy in resting HEK293 cells expressing 5-LO and FLAP revealed a homogenous intranuclear staining of 5-LO. Upon A23187 activation, 5-LO rapidly (within 90 s) translocated to the nuclear membrane and colocalized with FLAP. As shown in Fig. 4D, only CI was able to block A23187-induced 5-LO translocation in HEK293 cells expressing 5-LO and FLAP, while CS was not successful. These data additionally support the hypothesis on CI activity in blocking not only 5-LO enzymatic activity and upstream signalling but also the critical intracellular trafficking events needed for LT biosynthesis.

### 3.6 Effects of litreol derivatives on cyclooxygenase activity and PGE_2_ formation

**Fig. 6:**
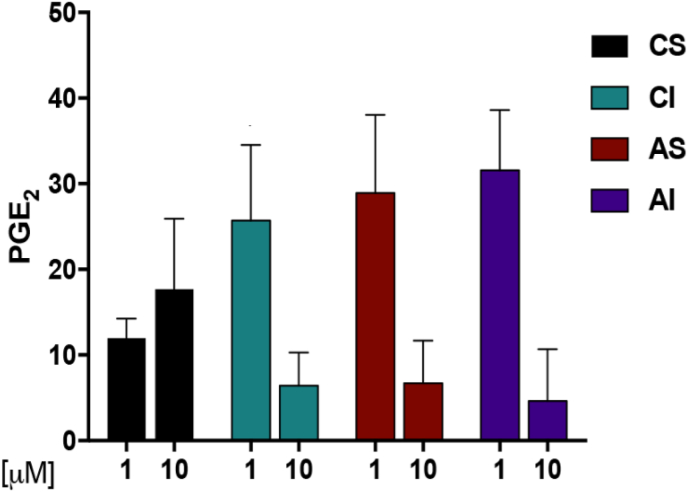
Selected values of PGE_2_ biosynthesis formed by monocyte. Monocytes treated with 1 and 10 μM 1, 2, 3 and 4, were incubated by addiction of 10 g/ml lipopolysaccharide and AA substrate necessary for PGE_2_. Formed pro-inflammatory eicosanoid were isolated by solid –phase extraction and analyzed by LC-MS/MS. Results are given as means ± S.E.M., n = 4.

We extended our investigation on the evaluation of litreol compound interference with other key enzymes involved in eicosanoid biosynthesis. As LTs and PGs arise from the same substrate, AA, it is crucial to assess whether these compounds also modulate COX enzyme activity responsible for prostaglandin production. To this aim, we used human isolated monocytes, a major source for LTs biosynthesis [25], as cell-based assay system. We evaluated if CI, and its derivatives (CS, AI and AS) may affect also COX-mediated PGE_2_ production. Monocytes were stimulated with LPS or A23187 and, as showed in Fig. 6, the treatment with all four compounds at 1–10 μM significantly inhibited PGE_2_ formation.

### 3.7 Binding mode investigation into 5-LO and in silico ADME profiling

**Fig. 7:**
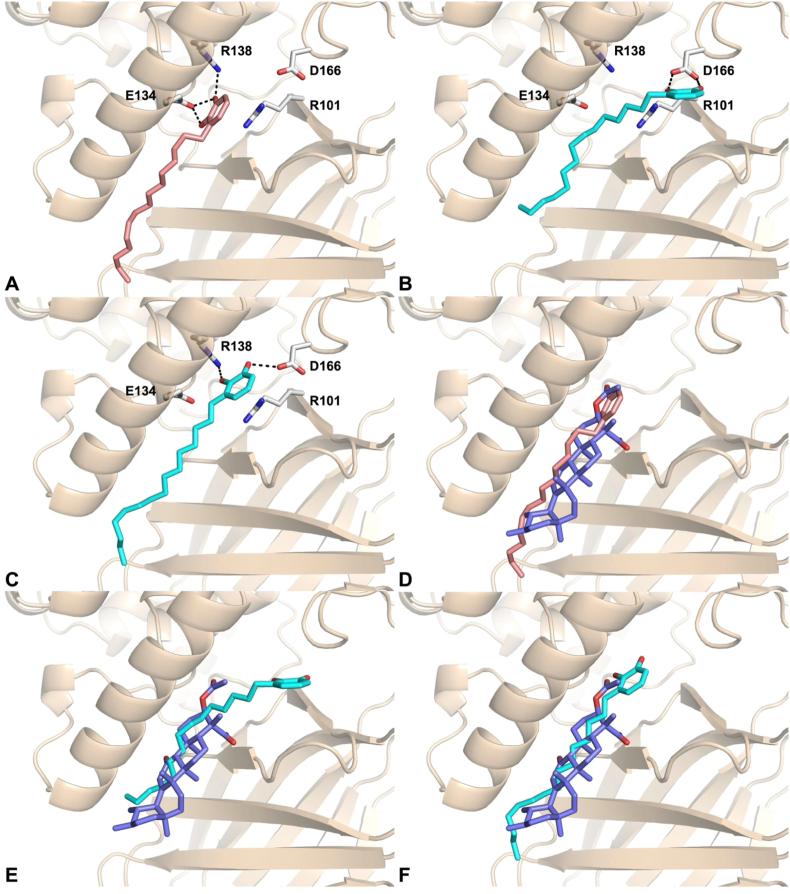
Binding mode of compounds CI(A, salmonsticks), CS pose 1 (B, cyan sticks), CS pose 7(C, cyan sticks), into 5-LO allosteric site (wheat ribbons, PDB 6NCF) as predicted by docking calculations. Overlay of CI(D), CS pose 1 (E) and CS pose 7 (F) with AKBA (slate sticks).Only amino acid residues discussed in the main text are displayed (white sticks) and labeled. Hydrogen bonds discussed in the text are depicted as dashed black lines.

**Fig. 8:**
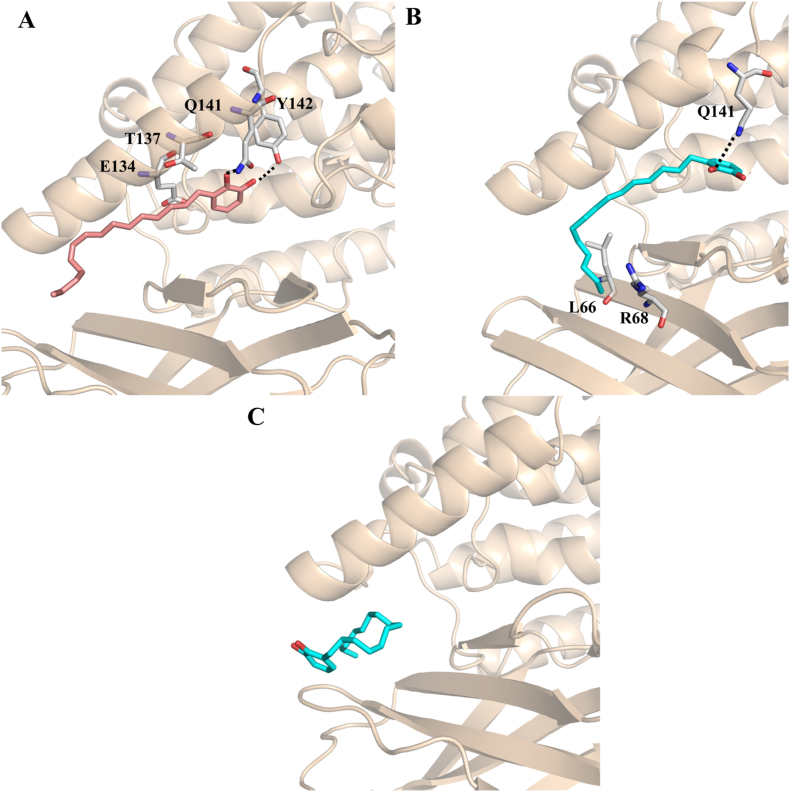
Most representative centroid configurations for compounds CI (A, salmon sticks), CS pose 1 (B, cyan sticks), CS pose 7 (C, purple sticks), into 5-LO allosteric site (wheat ribbons, PDB 6NCF) as extracted from MD simulations. Only amino acid residues discussed in the main text are displayed (white sticks) and labeled. Hydrogen bonds discussed in the text are depicted as dashed black lines.

In order to clarify the possible binding mode of the synthesized compounds, we undertook docking studies using the Glide module (Glide, Schrödinger, LLC, New York, NY, 2023) [33,34] which is part of the Maestro software suite. The 5-LO enzyme consists of two main domains: an N-terminal region containing two four-stranded antiparallel β-sheets, known as the PLAT (Polycystin-1, Lipoxygenase, Alpha-Toxin) domain [40] and a C-terminal catalytic domain that harbors a non-heme iron. A recently identified allosteric site, situated at the interface between the PLAT and catalytic domains, has been shown to accommodate small molecules such as the natural product 3-acetyl-11-keto-beta-boswellic acid (AKBA), which functions as a non-competitive inhibitor. In contrast, the competitive inhibitor nordihydroguaiaretic acid (NDGA) binds directly within the catalytic pocket, leading to pronounced structural disorder in helix α2, which borders the active site. Compounds CI and CS were docked into the allosteric binding site of 5-LO, based on experimental evidence indicating that neither compound competes with arachidonic acid (AA) for the active site. Docking calculations were performed using the crystal structure of 5-LO (PDB: 6NCF) in complex with AKBA, generating and analyzing ten poses for each compound. The binding mode of CI revealed key interactions that partially overlap with those of AKBA [32]. Notably, CI engages in a π-stacking interaction with R101 and forms hydrogen bonds with R138 via one of the catechol hydroxyl groups. Additionally, the side chain of E134 appears to be engaged by both hydroxyl groups of the catechol moiety, further stabilizing the complex (Fig. 7A). The overlay of the docked pose of CI with AKBA (Fig. 7D) revealed a clear spatial overlap between the alkyl chain of CI and the triterpene scaffold of AKBA. Additionally, the hydroxyl group of CI aligned closely with the carboxylate moiety of AKBA, suggesting a potential mimicry in key interaction points within the allosteric site. In contrast, CS, which lacks the double bond present in CI, was able to penetrate more deeply into the allosteric pocket. In its top-ranked pose (pose 1), CS lost interactions with both arginine residues, instead forming a strong hydrogen bond network with D166 through its catechol hydroxyls (Fig. 7B). However, an alternative binding mode (pose 7) was also identified, in which CS adopted a conformation more closely resembling that of CI and AKBA (Fig. 7C and F). In this pose, CS re-established key interactions with Arg101 through π-stacking and with Arg138 via one of the catechol hydroxyl groups, while also forming an additional hydrogen bond with Asp166 through the second hydroxyl. These results suggest that both CI and CS can target the allosteric site of 5- LO, though with slightly different binding modes and key interaction profiles. The binding modes obtained by docking studies helped also to rationalize the structure-activity relationships. In fact, CI demonstrated higher inhibitory activity against 5-LO (IC_50_ = 0.06 μM) compared to CS (IC_50_ = 0.16 μM), likely due to its ability to form a more extensive network of interactions comprising R101, R138, and E134 within the allosteric pocket, facilitated by its catechol moiety and the presence of the double bond, which contributes to optimal ligand conformation and positioning. CS, lacking the unsaturation, was able to access deeper regions of the pocket but showed reduced interaction stability, consistent with its slightly lower potency. The acetylated derivatives AS and AI were completely inactive in cell-free assays (IC_50_ & gt; 10 μM), which can be attributed to the masking of the catechol hydroxyl groups, representing key pharmacophores for hydrogen bonding with critical residues such as R138, R101, and R166. These results highlight the importance of the free catechol motif and the overall molecular conformation in achieving high-affinity binding and effective inhibition of 5-LO through allosteric modulation. The complexes obtained by docking studies were subjected to molecular dynamics (MD) simulations of 300 ns. These simulations allowed evaluation of both the persistence of key ligand-protein contacts and the overall conformational stability of the binding modes. The RMSD of backbone atoms of 5-LO and of the bound ligands in the allosteric sites were computed for all systems (Fig. s1). The RMSD values of both backbone atoms of 5-LO remained rather constant during the simulations, indicating there are no notable conformational changes. Deviations from the starting configuration were observed for CI, CS pose 1 and CS pose 7, due to the translational ligand rearrangement in the pocket. However, within the last 50 ns their RMSD values do not present significant change, indicating the stabilization of the ligands in the pocket. We employed an unsupervised ML clustering analysis to identify dissimilarities in binding arrangements over time. This approach aims to provide a comprehensive picture of the binding modes. As descriptors, we collected all atoms coordinates for each system. We then reduced for each simulation the data complexity using PCA analysis, preserving 90 % of the total variance. As a result, we obtained four principal components for CI and CS pose 1, while five components for CS pose 7. The subsequent clustering on the PCA matrices returned 4 clusters as best choices for CS pose 7 (Fig. S2C), while three clusters were found for CS pose 1 and CI (Fig. S2A and B). Below, we describe the binding modes observed following the molecular dynamics (MD) simulations and clustering analysis. For each compound, the representative structure from the most populated cluster was selected for further examination. In the case of compound CI, the catechol moiety established new hydrogen bonds with Q141 and Y142 side chain, while the alkyl chain's movement appeared to be stabilized by hydrophobic interactions with apolar side-chain carbons of T137 and E134 (Fig. 8A). For compound CS, pose 7 did not exhibit any notable interactions, likely due to the ligand drifting away from the allosteric site (Fig. 8C). In contrast, pose 1 revealed the formation of a hydrogen bond (3.1 Å) between one of its hydroxyl groups and Q141 (Fig. 8B). Additionally, hydrophobic interactions between the alkyl chain and residues R68 and L66 (located on the β-sheets) contributed significantly to the binding stability. Collectively, these results underscore that durable allosteric inhibition of 5-LOX by this class of inhibitors requires a cooperative network interaction, where the Q141 residue plays an important role.

To complement the binding mode analysis of the selected compounds with 5-LO, their physicochemical and pharmacokinetic properties were predicted using SwissADME [Ref: SwissADME: a free web tool to evaluate pharmacokinetics, drug-likeness and medicinal chemistry friendliness of small molecules [41]. A selection of key descriptors is reported in Table S1, to provide insights into their drug-likeness and pharmacokinetic potential. The results show that the compounds generally fall within the accepted boundaries of drug-like chemical space in terms of MW, lipophilicity, TPSA. Nonetheless, predictions indicate potential issues in aqueous solubility, which may impact their oral bioavailability.

## 4 Discussion

Our comprehensive pharmacological investigation of synthetic litreol derivatives, proposed CI (3-(pentadec-10′-enyl)-catechol) as most promising 5-LO inhibitor and other key enzymes involved in eicosanoid biosynthesis. Using isolated human polymorphonuclear leukocytes and monocytes as physiologically relevant cell models, we demonstrated that these compounds effectively inhibit leukotriene and prostaglandin production through multiple complementary mechanisms. Consistent with their prominent suppression of 5-LO product formation, CI and its derivatives demonstrated significant inhibitory effects in both cellular and cell-free assays, confirming direct action on 5-LO enzymatic activity. Besides CI also the derivative CS was efficient, and both compounds also suppressed 12-LO. Zileuton is the only approved 5-LO inhibitor that requires high dosing [27], is an unspecific suppressor of PG biosynthesis [42,43], and induces hepatotoxic side effects in patients [44]. However, its clinical utility is limited due to the requirement of high dosing, non-specific suppression of prostaglandin biosynthesis, and hepatotoxic side effects. These limitations underscore the urgent need to develop more potent and safer 5-LO inhibitors with improved pharmacological profiles for therapeutic use. Therefore, there is a strong need to develop more potent and safer 5-LO inhibitors with favourable pharmacological profile for therapeutic use [27]. In this context, several natural compounds have been identified as modulators of 5-LO and related pathways. Notably, quercetin and curcumin have been shown to inhibit 5-LO activity and leukotriene production in various inflammatory models [45]. Nordihydroguaiaretic acid (NDGA) is a well-known lipoxygenase inhibitor that acts broadly on 5-LO and 12-LO [46] and has been extensively studied for its anti-inflammatory and antioxidant properties. Cirsiliol, a flavonoid compound, was reported to suppress LTB4 biosynthesis and inhibit 5-LO translocation. Moreover, panaxynol, a polyacetylene derived from *Panax ginseng*, has shown inhibitory effects on 5-LO product formation and ROS generation. Similarly, caffeic acid and sinapic acid, both hydroxycinnamic acid derivatives, exert anti-inflammatory effects in part through interference with LOX and COX pathways [47]. Caffeic acid phenethyl ester (CAPE), a bioactive compound found in propolis, has been investigated as a potential 5-lipoxygenase (5-LO) inhibitor. CAPE exhibits significant anti-inflammatory activity, with an IC_50_ of 0.5 μM in isolated human neutrophils—lower than that of the approved drug zileuton. However, its efficacy decreases under physiological conditions (IC_50_ of 1.8 μM in whole blood), likely due to hydrolysis by esterases, which convert CAPE into caffeic acid, a compound with poor inhibitory activity against 5-LO [48]. We investigated the effects of CI and of its synthetic derivatives on 5-LO product synthesis in a *cell-free* assay using isolated human recombinant 5-LO and in a *cell-based* assay using human PMNL. The *cell-free* assay enables the identification of compounds that directly interfere with 5-LO activity, whereas the cell-based assay system considers various cellular regulatory aspect of 5-LO product synthesis as well. The inclusion of the cell-free assay was critical, as 5-LO activity is known to be sensitive to redox regulation and requires a precise peroxide threshold. This suggested that litreol derivatives might also affect reactive oxygen species (ROS) formation, which we confirmed by showing their interference with ROS generation in PMNL upon PMA stimulation. The *cell-based* assay offers several possible points of attack of a given compound such as blockade of 5-LO-activating protein (FLAP) or coactosine-like protein (CLP), interference with lipid hydroperoxides, protein kinases, Ca^2+^ mobilization, and 5-LO translocation. CI and CS exhibited potent inhibition of 5-LO product synthesis in PMNL (IC_50_ of 0.26 and 0.8 μM, respectively) as well as in both intact cells and *cell-free* assays (IC_50_ 0.06 and 0.15 μM, respectively). Therefore, CI and CS exhibited very low IC_50_ values as compared to the acetylated derivatives AS and AI in intact cells (IC_50_ up to 0.5 μM) as well as in cell-free assays (IC_50_ > 10 μM). The mechanism by which CI inhibited 5-LO activity seems to be non-competitive as the increase of substrate concentration did not decrease the efficiency of the compounds. Notably, the litreol compounds were not cytotoxic on PMNL, as assessed by LDH assay.

Our investigations into upstream signalling pathways revealed differential effects of the compounds on p38 MAPK phosphorylation and intracellular calcium mobilization, two key regulators of 5-LO activation. Notably, CI compound inhibited fMLP-induced p38 MAPK activation, whereas CS did not, and neither compound altered intracellular Ca^2+^ levels. This differential modulation indicated distinct mechanisms of action among the derivatives, with CI potentially targeting signalling cascades that can amplify 5-LO activity. Moreover, Western blot analyses under LPS stimulation showed a strong inhibition of NF-κB expression by all derivatives and again CI was the most potent. This suggests that litreol derivatives may also exert anti-inflammatory effects by selectively suppressing transcription factors like NF-κB, thereby modulating the expression of pro-inflammatory genes either independently or downstream MAPK signalling.

Besides the absolute supply of endogenous substrate for 5-LO, the accessibility of AA is another crucial event in 5-LO product formation of intact cells and depends on the transfer of AA from the nuclear membrane-bound FLAP to 5-LO. FLAP inhibitors efficiently block 5-LO product formation in intact cells by impeding 5-LO/FLAP complex formation at the nuclear envelope and thereby interrupting AA transfer from FLAP to 5-LO. FLAP is located at both the nuclear membrane and ER of leukocytes and is essential for 5-LO product formation from endogenous AA in intact cells [29]. Upon cell activation, 5-LO translocates from the cytosol or the nucleoplasm to the perinuclear region and interacts with FLAP. HEK293 cells stably transfected for co-expression of FLAP and 5-LO were monitored for 5-LO nuclear translocation and for the capacity to generate 5-LO products. CI, but not CS, suppressed 5-LO translocation in activated cells thereby preventing the co-localization with FLAP to receive AA as substrate. It also appeared possible that CI and CS could interrupt signalling pathways that are required for 5-LO activation, including Ca^2+^ mobilization, ROS formation, and phosphorylation events. However, ROS formation and Ca^2+^ mobilization in activated PMNL were not affected by CI and CS, while p38 MAP activation was suppressed by CI. We investigated whether CI and CS may affect also other enzymes involved in eicosanoid biosynthesis and we found that CI and CS repressed 12-LO activity (IC_50_ 3.15 μM and 5.10 μM, respectively). Extending our analyses beyond LTs, we also assessed the impact of litreol derivatives on COX-mediated PGE_2_ formation in human monocytes stimulated with LPS or A23187. All compounds, except for CS, significantly reduced PGE_2_ production in LPS-stimulated cells, indicating effective inhibition of COX activity under inflammatory conditions. The dual inhibition of 5-LO and COX pathways induced by some litreol derivatives and above all CI highlights the therapeutic potential of these compounds as broad-spectrum modulators of eicosanoid-mediated inflammation.

Based on the computational studies, the observed differences in binding modes and stability of CI and CS within the allosteric site of 5-LO provide a rational explanation for their divergent inhibitory activities. The stronger interaction network of CI likely contributes to its superior potency. In contrast, the increased flexibility and reduced interaction stability of CS correlate with its lower activity. Moreover, the critical role of the free catechol group in mediating key interactions was reinforced by the inactivity of acetylated derivatives.

Implications for lead optimization emerge from our docking and MD analyses, which indicate that high-affinity allosteric inhibition relies on a catechol-centered network of interactions, a feature that should be preserved in future derivatives. To enhance potency, structural modifications will focus on constraining the hydrophobic tail to favor a CI-like conformation, for example by preserving or introducing an E-alkene, adding gem-dimethyl substitution, or embedding a cyclopropyl unit to reduce flexibility and stabilize the preferred binding arrangement. In parallel, the limited solubility predicted in silico could be mitigated by introducing heteroatoms within the hydrophobic tail (e.g., –CH_2_–O–CH_2_– or –CH_2_–CONH–) or by attaching small ionizable groups such as N,N-dimethylamino at the distal end of the molecule. These modifications are expected to improve solubility and overall drug-likeness while preserving the essential catechol pharmacophore and its key interactions within the allosteric pocket.

## 5 Conclusions

5-LO activity inhibition reduces pro-inflammatory LT formation, representing a valid drug target for the pharmacotherapy of inflammatory disorders and for some cancers. To date, although zileuton is the only 5-LO inhibitor currently in clinical use, it is required to develop novel LT biosynthesis inhibitors with improved potency and safety. Therefore, the high efficiency of CI and CS against 5-LO is encouraging and supports their potent anti-inflammatory effectiveness. Moreover, we demonstrated that the natural catechol compound CI and its derivative CS are highly selective and highly potent 5-LO inhibitors in intact human leukocytes. Our results shed light on the mechanism of 5-LO activity inhibition with CI acting as a direct non-competitive 5-LO inhibitor. In this context, CI was also able to prevent the 5-LO/FLAP interaction and interrupted signalling pathways that are required for 5-LO activation such as p38 MAP kinase activation. The structurally related AS and AI did not show encouraging results on inhibition of 5-LO. Importantly, we emphasize that the hydrogenation of the double bond in litreol (resulting in the compound CS) significantly reduces its inhibitory activity; comparison of IC_50_ values indicates that CI is approximately 2.5 to 3-fold more potent than its hydrogenated analogue CS. Together, we reveal CI and CS as novel and potent 5-LO inhibitors with potential use for treatment of LT-related inflammatory and allergic disorders. Our computational modeling revealed that high-affinity binding of CI is driven by a catechol-centered hydrogen bond network and the optimal positioning of the hydrophobic chain. Future optimization efforts will focus on stabilizing the hydrophobic chain, improving solubility, and enhancing pharmacokinetics without compromising binding. Additionally, CI's dual mechanism of action opens avenues for broader therapeutic applications, including selective modulation of the leukotriene and prostaglandin pathways. Future studies are warranted to elucidate the precise molecular targets of these compounds and to evaluate their efficacy and safety in in vivo models of inflammation.

Moreover, the design of next generation derivatives will focus on preserving the catechol pharmacophore while introducing constraints within the hydrophobic tail and distal polar groups to improve both potency and solubility.

## Author contributions

R.F. and O.W. contributed to the conceptualization of the study. A.M.C. performed the in vitro experiments and was involved in data interpretation and curation. F.B. conducted the synthesis experiments and contributed to manuscript writing. L.A., F.B., and R.A.C.M. were responsible for the design of the synthetic procedures, methodology development, data curation, and validation. S.P. designed the in vitro study. M.C., S.Z., O.W., and R.F. provided supervision and manuscript editing. M.C., P.A., O.W., and R.F. secured funding for the project.

## Funding

This research was partially supported by the “Ricerca Corrente” funding scheme of the Ministry of Health, Italy and by the POR CAMPANIA FESR 2014–2020 Asse Prioritario 3–Progetto Microactive-Dominio tecnologico-produttivo.

## Declaration of competing interest

The authors declare that they have no known competing financial interests or personal relationships that could have appeared to influence the work reported in this paper.

## Data Availability

Data Availability Statement: The datasets generated and analyzed during the current study are available in the Supplementary Materials.

## List of abbreviations

**AA**: arachidonic acid
**AI**: 1,2-diacetyl-3-(pentadec-10′-enyl)-benzene
**AS**: 1,2-diacetyl-3- penta-decylbenzene
**ATP**: adenosine triphosphate
**CI**: 3-(pentadec-10′-enyl)-catechol
**COX**: cyclooxygenase
**cPLA2**: cytosolic phospholipase
**CS**: 3-pentadecylcatechol
**DAPI**: 4′,6-diamidino-2-phenylindole, dihydrochloride
**DMAP**: 4-dimethylaminopyridine
**DMSO**: dimethyl sulfoxide
**EDTA**: ethylenediaminetetraacetic acid
**fMPL**: synthetic formyl-peptide
**HEK-293**: human embryonic kidney
**5-HPETE**: 5-hydroperoxyeicosatetraenoic acid
**5-HpETE**5(S): hydroper-oxy-6-trans-8,11,14-cis-eicosatetraenoic acid
**FCS**: fetal calf serum
**IL-1β**: interleukin-1beta
**LTs**: leukotrienes
**LTA4**: leukotriene A4
**LTB4**: leukotriene B4
**5-LO**: 5-Lipoxygenase
**12-LO**: 12-lipoxygenase
**FLAP**: 5-lipoxygenase-activating protein
**MAP**: membrane associated proteins in eicosanoid and glutathione metabolism
**MAPK**: mitogen-activated protein kinases
**NADH**: nicotinamide adenine dinucleotide
**NF-κB**: nuclear factor kappa-light-chain-enhancer of activated B cells
**Nrf2**: nuclear factor erythroid 2–related factor 2
**PBS**: phosphate buffered saline
**PG buffer**: PBS-glucose buffer
**PGC buffer**: PBS-glucose-Ca^+2^ buffer
**PMA**: phorbol myristate acetate
**PMNL**: polymorphonuclear leukocytes
**PGE_2_**: prostaglandin E_2_
**ROS**: oxygen reactive species
**SDS**: sodium dodecyl-sulfate
**SPM**: specialized pro-resolving mediators
**TNFα**: tumor necrosis factor alfa
