# Corrigendum to “Evaluation of molecular mechanisms of (*Z*)-3-(pentadec-10′-enyl)-catechol (litreol) and synthetic derivatives as inhibitors of human leukotriene biosynthesis” [Redox Biol. 87 (2025) 103880]

**DOI:** 10.1016/j.redox.2026.104197

**Published:** 2026-05-23

**Authors:** Alessia Maria Cossu, Simona Pace, Ferdinando Bruno, Lucia Abbatiello, Carmen Cerchia, Emanuele Falbo, Alejandra Catalina Muñoz Ramírez, Christian Kretzer, Laura Miek, Fabiana Troisi, Jana Gerstmeier, Pasquale Ambrosino, Silvia Zappavigna, Antonio Lavecchia, Oliver Werz, Michele Caraglia, Rosanna Filosa

**Affiliations:** a Laboratory of Precision and Molecular Oncology, Biogem Scarl, Institute of Genetic Research, Ariano Irpino, 83031, Italy; b Department of Precision Medicine, University of Campania “Luigi Vanvitelli”, Via L. De Crecchio, 7, Naples, 80138, Italy; c Institute of Pharmacy, Friedrich-Schiller-University Jena, Philosophenweg 14, Jena, D-7743, Germany; d Department of Science and Technology, University of Sannio, Benevento, 82100, Italy; e Department of Pharmacy, “Drug Discovery” Laboratory, University of Naples “Federico II”, Via D. Montesano 49, Napoli, 80131, Italy; f Department of Environmental Sciences, Faculty of Chemistry and Biology, University of Santiago de Chile, Santiago, 8320000, Chile; g Istituti Clinici Scientifici Maugeri IRCCS, Cardiac Rehabilitation Unit of Telese Terme Institute, Telese Terme, 82037, Italy; h Istituti Clinici Scientifici Maugeri IRCCS, Scientific Directorate of Telese Terme Institute, Telese Terme, 82037, Italy

The authors regret: The authors regret that the surname of the author Antonio La vecchia was incorrectly reported and should be corrected to Antonio Lavecchia. In addition, Fig. 5 contained a minor formatting error; the corrected figure is provided here. These corrections do not affect the results or conclusions of the paper. The authors would like to apologise for any inconvenience caused.

**Fig. 5:**
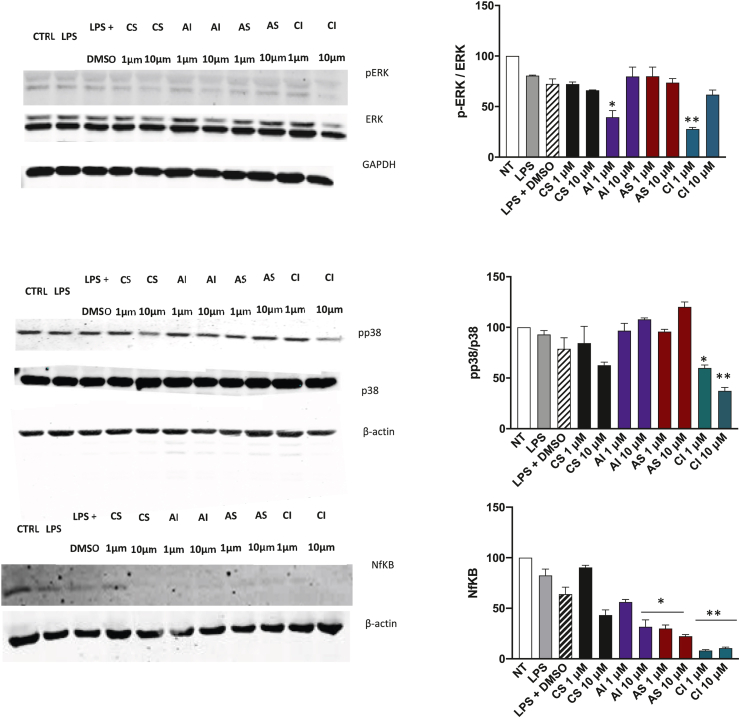
Modulation of p38-MAPK, ERK, and NF-κB by litreol compounds under pro-inflammatory stimuli. Western blot analysis of total and phosphorylated ERK (*p*-ERK), p38 MAPK, and NFκB in LPS-stimulated cells treated with different litreol derivatives. The experiments were repeated at least three times giving always similar results. Columns represent the intensity of the different bands evaluated as arbitrary units. Results are given as means ± S.E.M., n = 3.

The authors would like to apologise for any inconvenience caused.

