# Supplementary material for: Evaluation of molecular mechanisms of (Z)-3-(pentadec-10′-enyl)-catechol (litreol) and synthetic derivatives as inhibitors of human leukotriene biosynthesis
Source: Redox Biol. 2025 Sep 30;87:103880. doi: 10.1016/j.redox.2025.103880 (PMC12523804; doi:10.1016/j.redox.2025.103880)
Supplement: Multimedia component 1 [file mmc1.docx]

**Supporting Information**


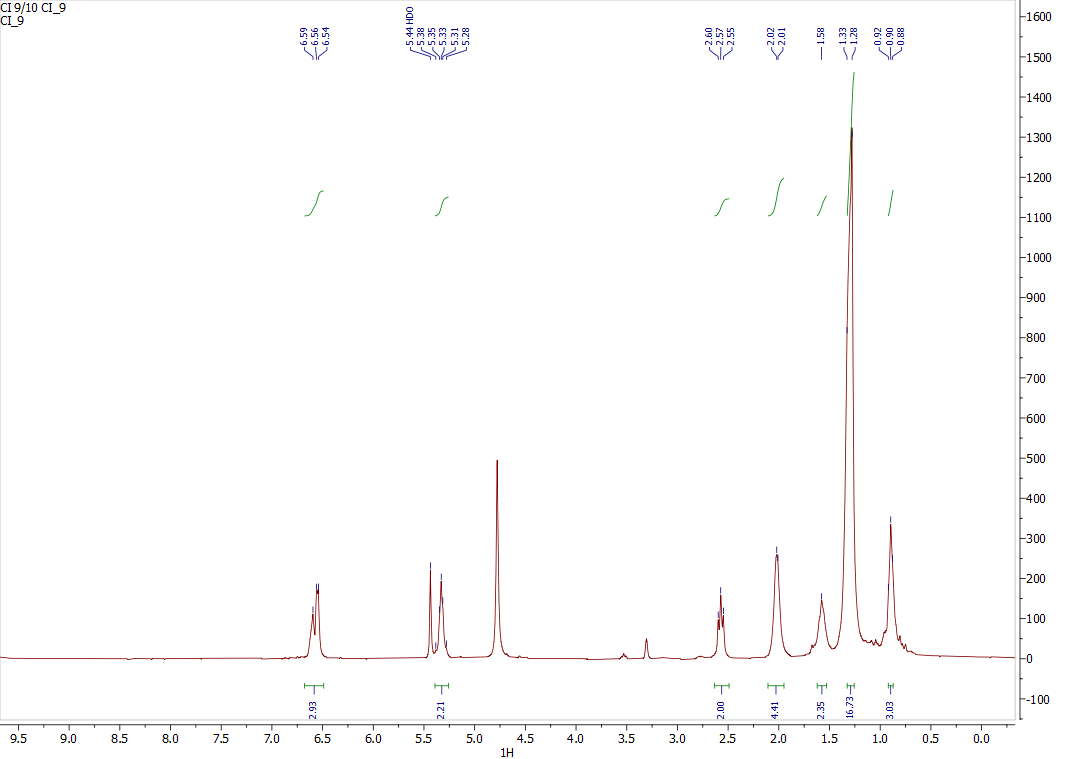
**^1^H NMR Spectra**

**CI**, CD_3_OD, 300 Hz

**^13^C NMR Spectra**

**^
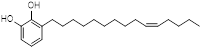
^**
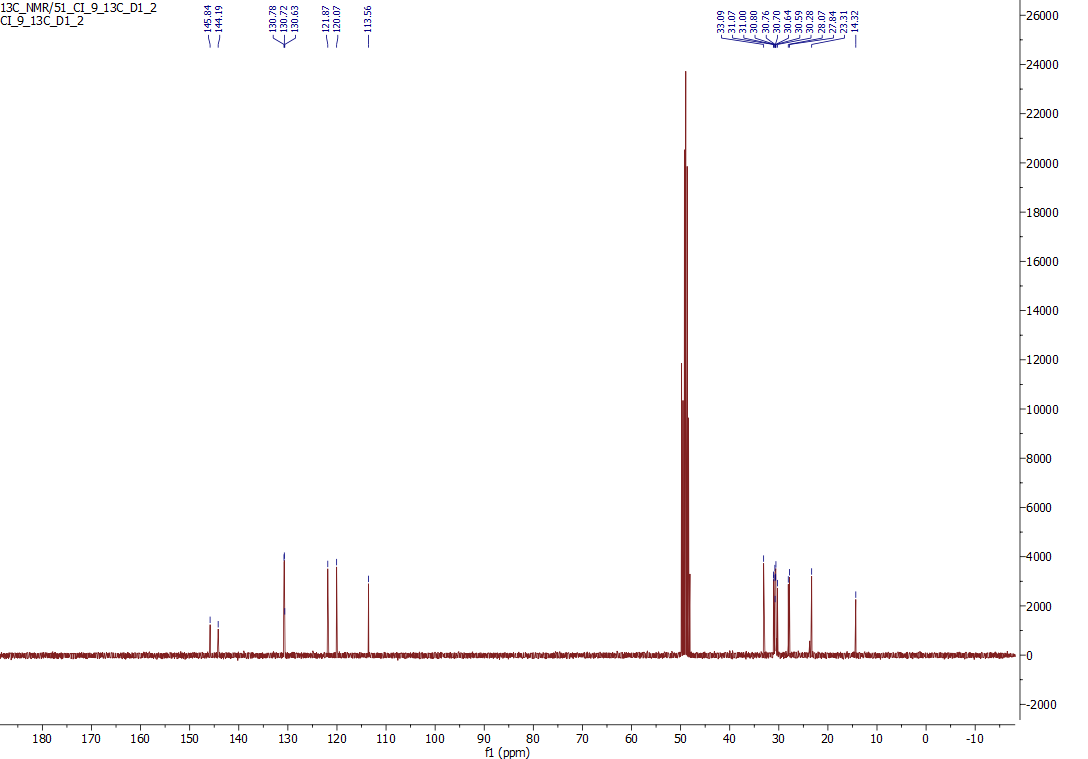


**CI**, CD_3_OD, 300 Hz

**^1^H NMR Spectra**


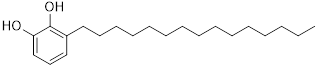

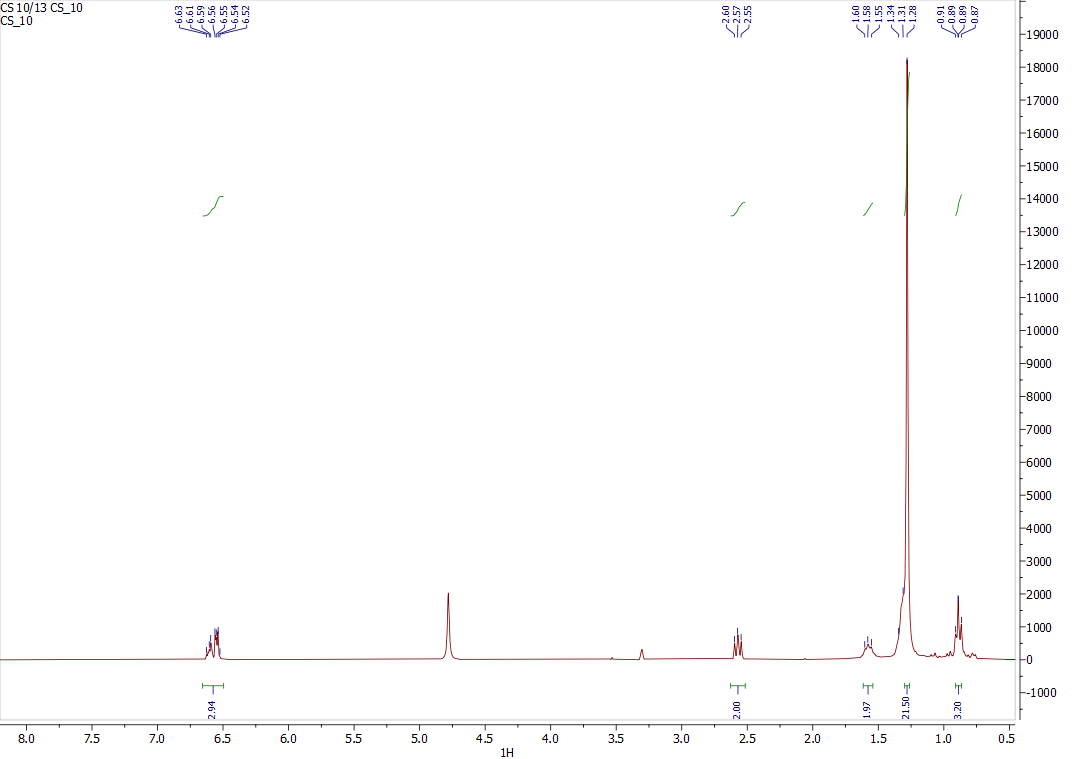


CS, CD_3_OD, 300 Hz

**^13^C NMR Spectra**

CS, CD_3_OD, 300 Hz


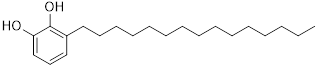

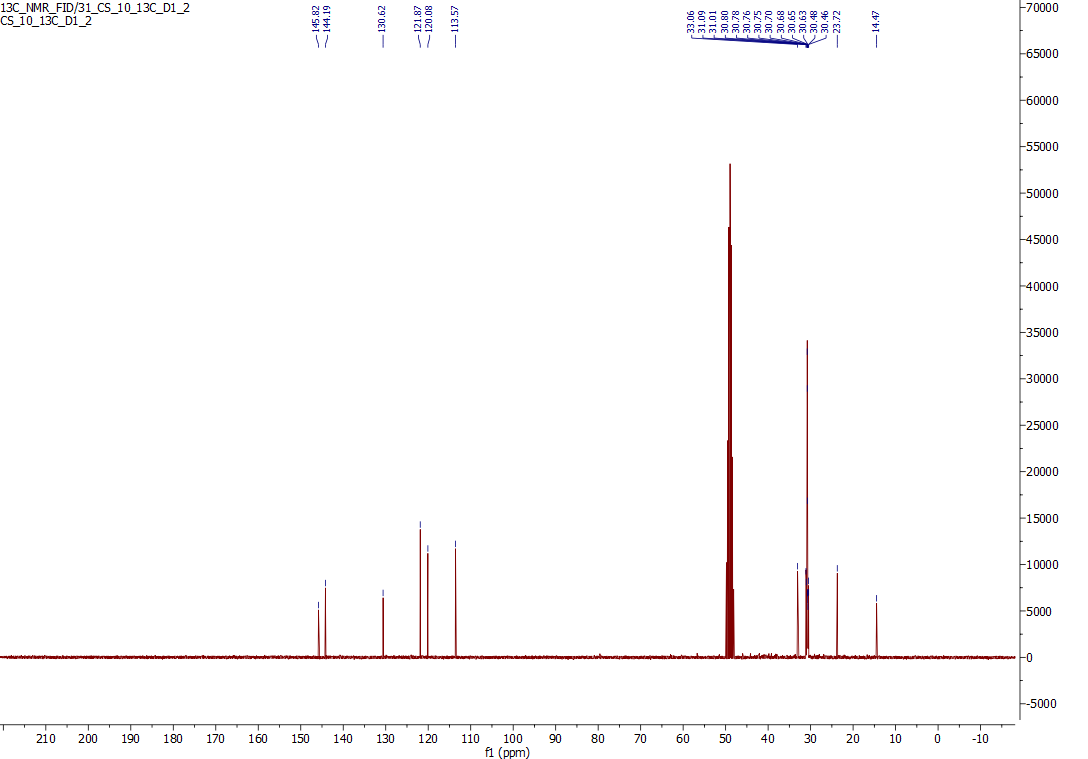


**^1^H NMR Spectra**


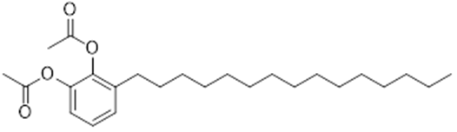

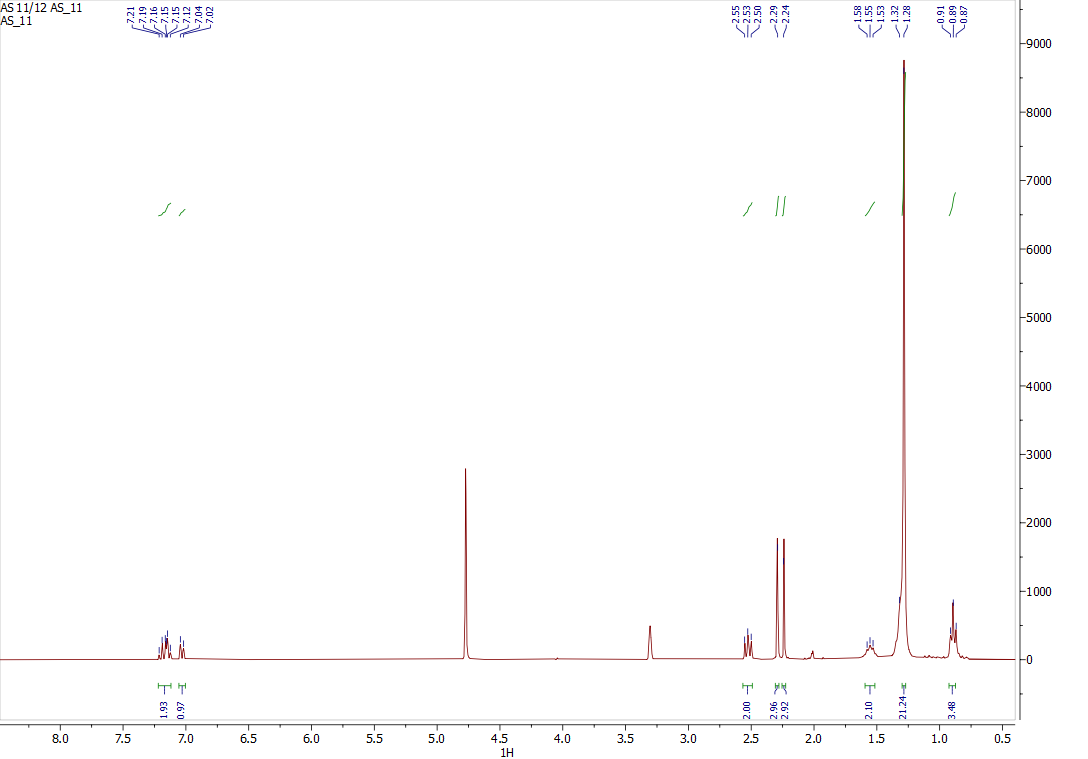


AS, CD_3_OD, 300 Hz

**^13^C NMR Spectra**

AS, CD_3_OD, 300 Hz


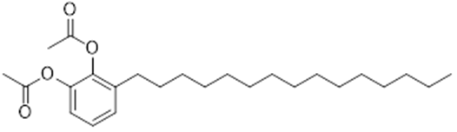

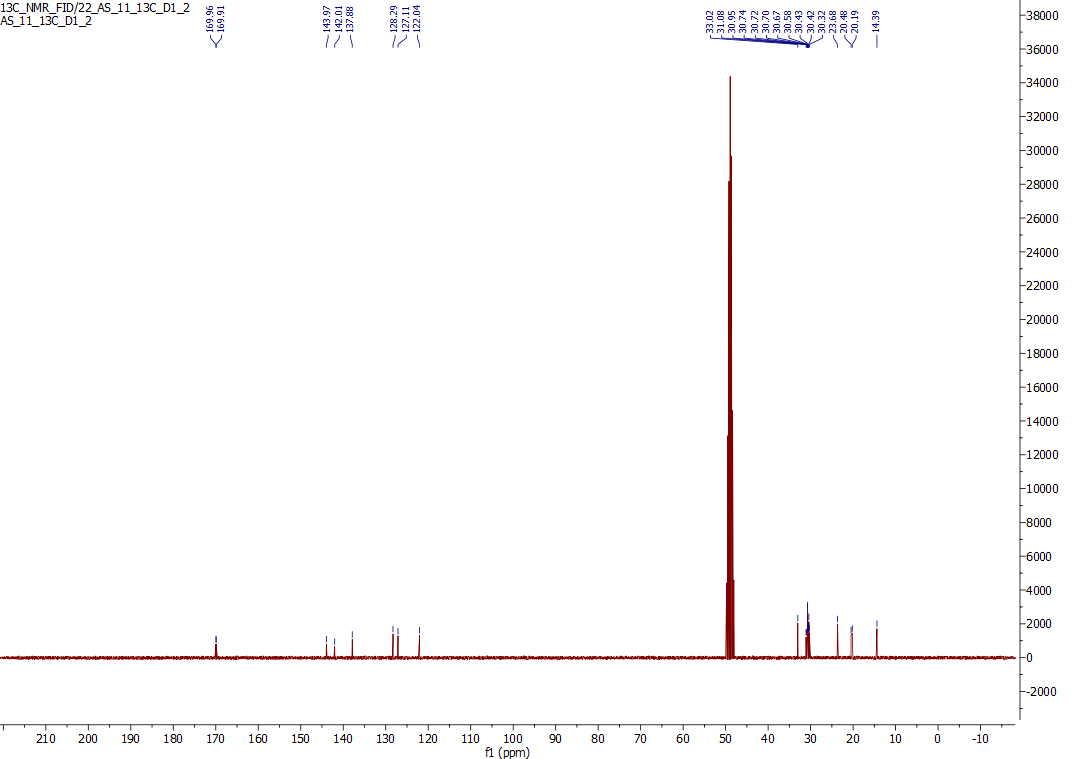


**^1^H NMR Spectra**


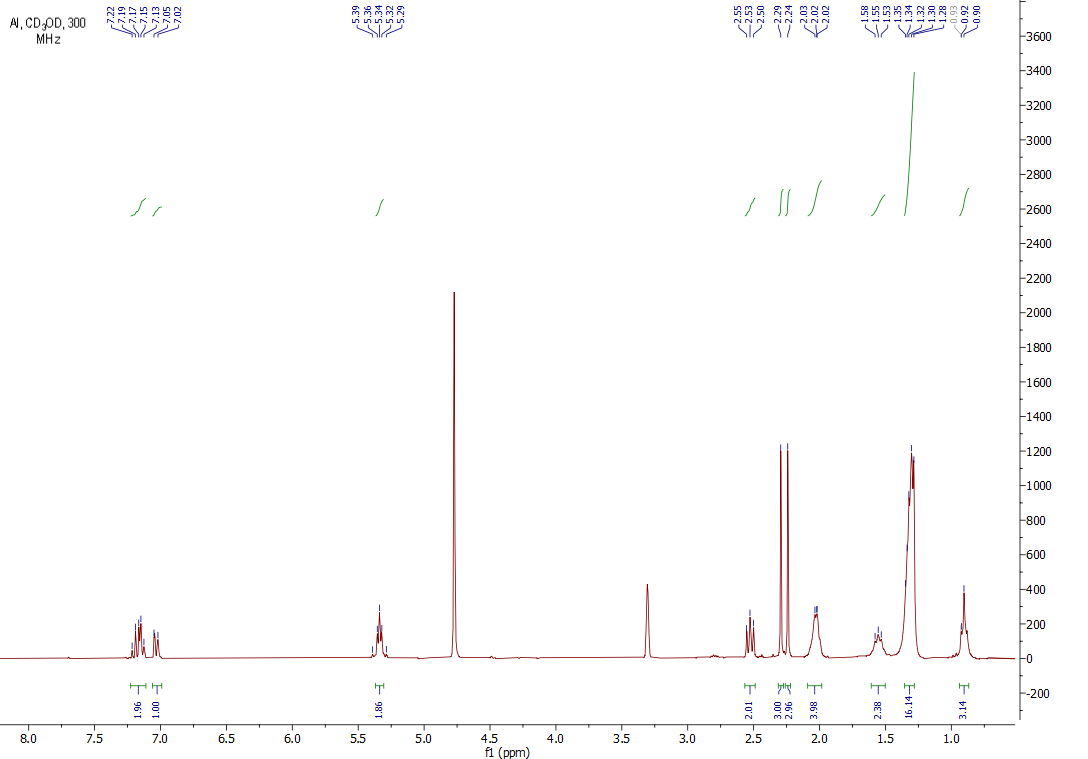


AI, CD_3_OD, 300 Hz

**^13^C NMR Spectra**

AI, CD_3_OD, 300 Hz


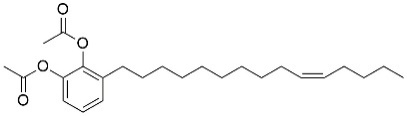

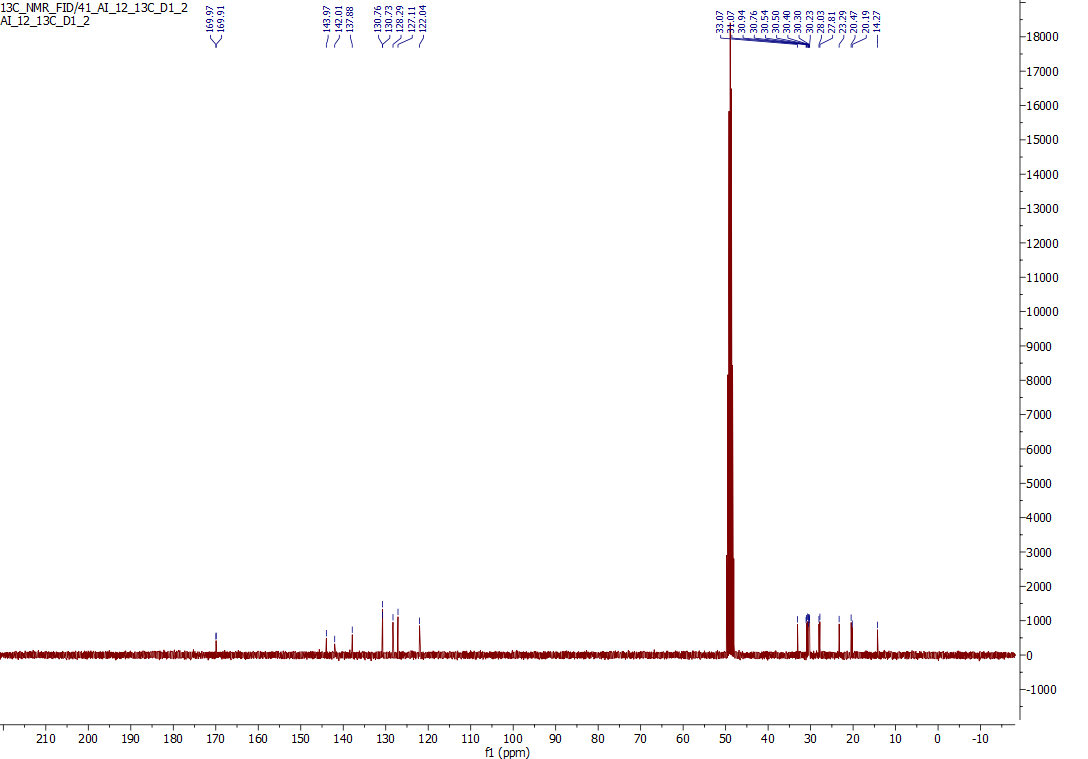


**COSY NMR Spectra**


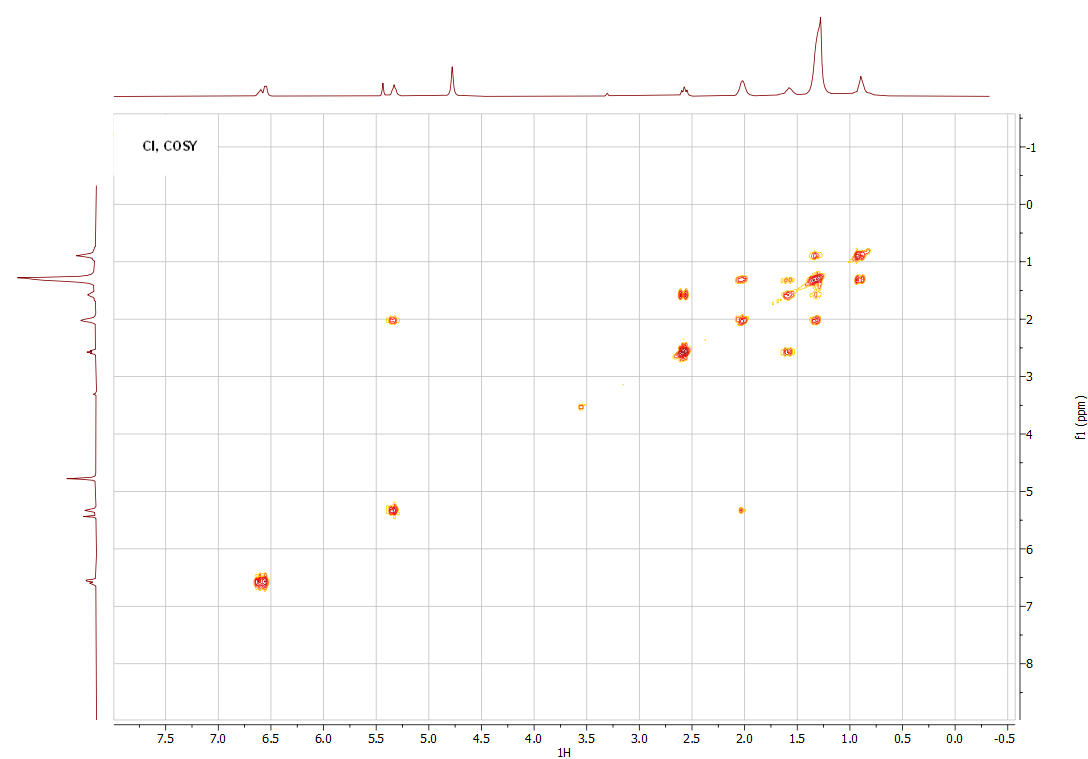


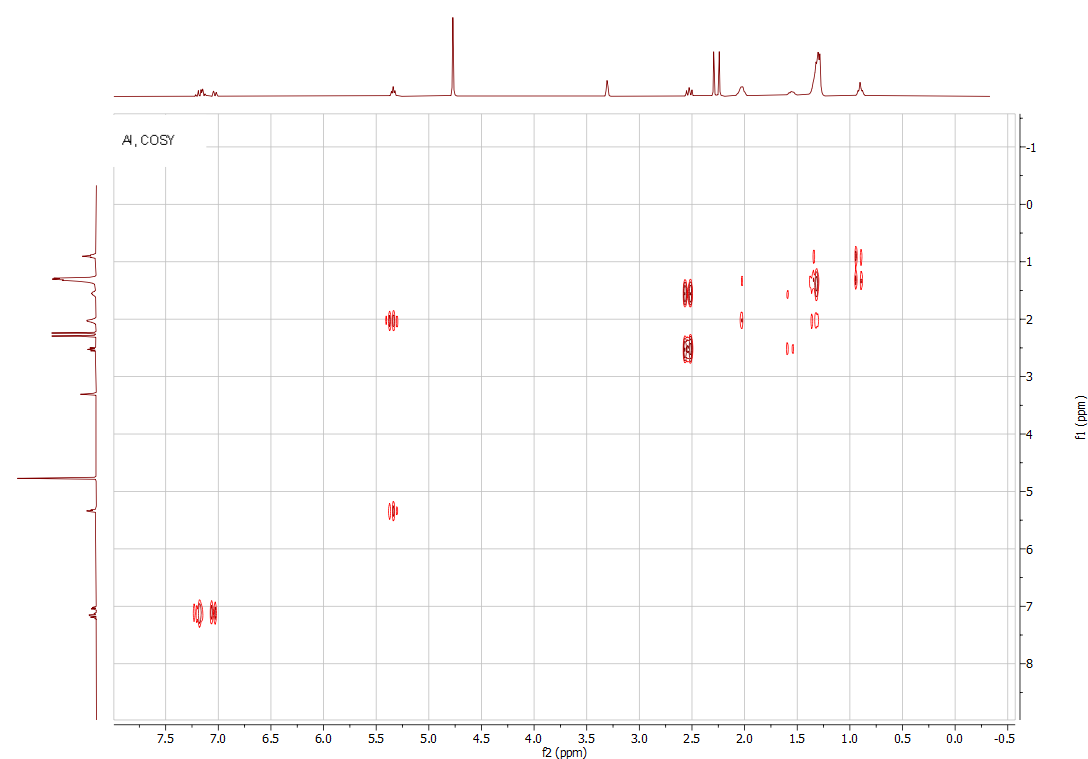


**HSQC NMR Spectra**


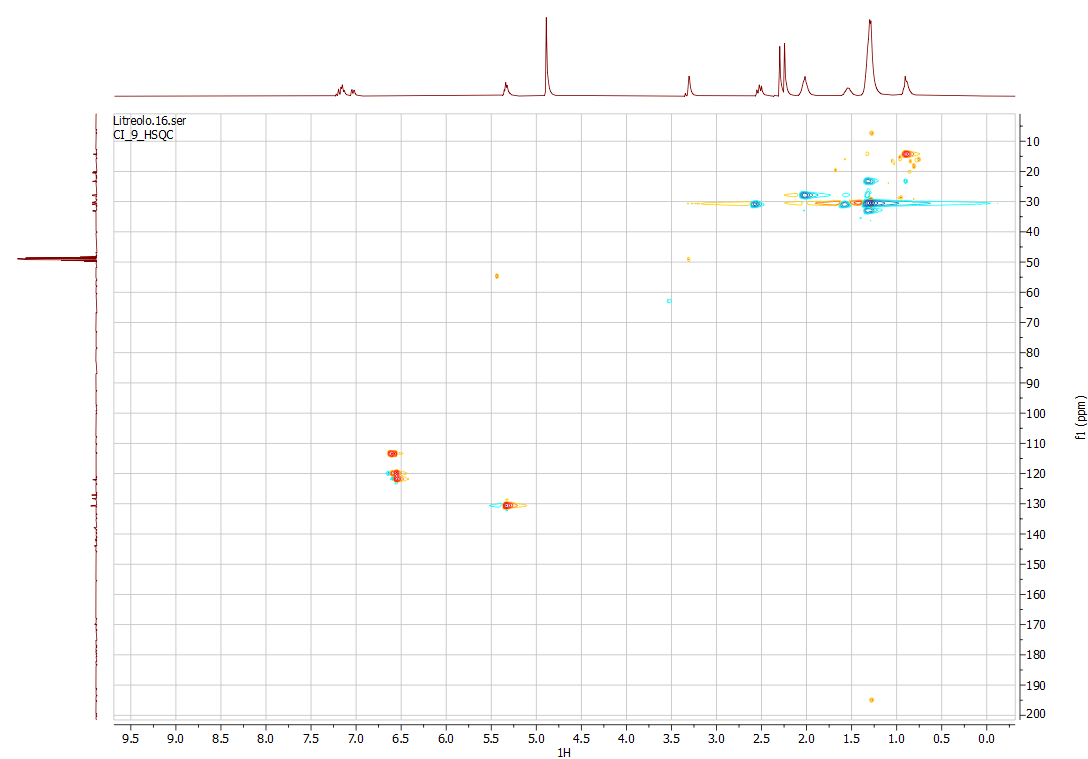


**H2BC NMR Spectra**


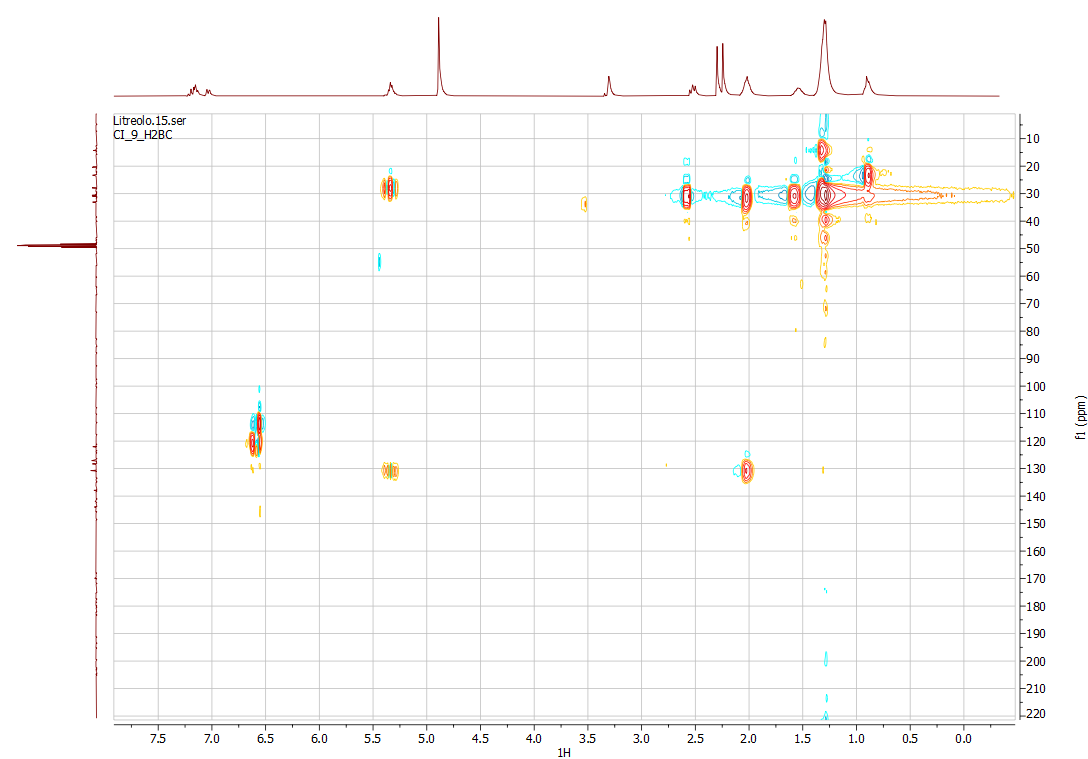


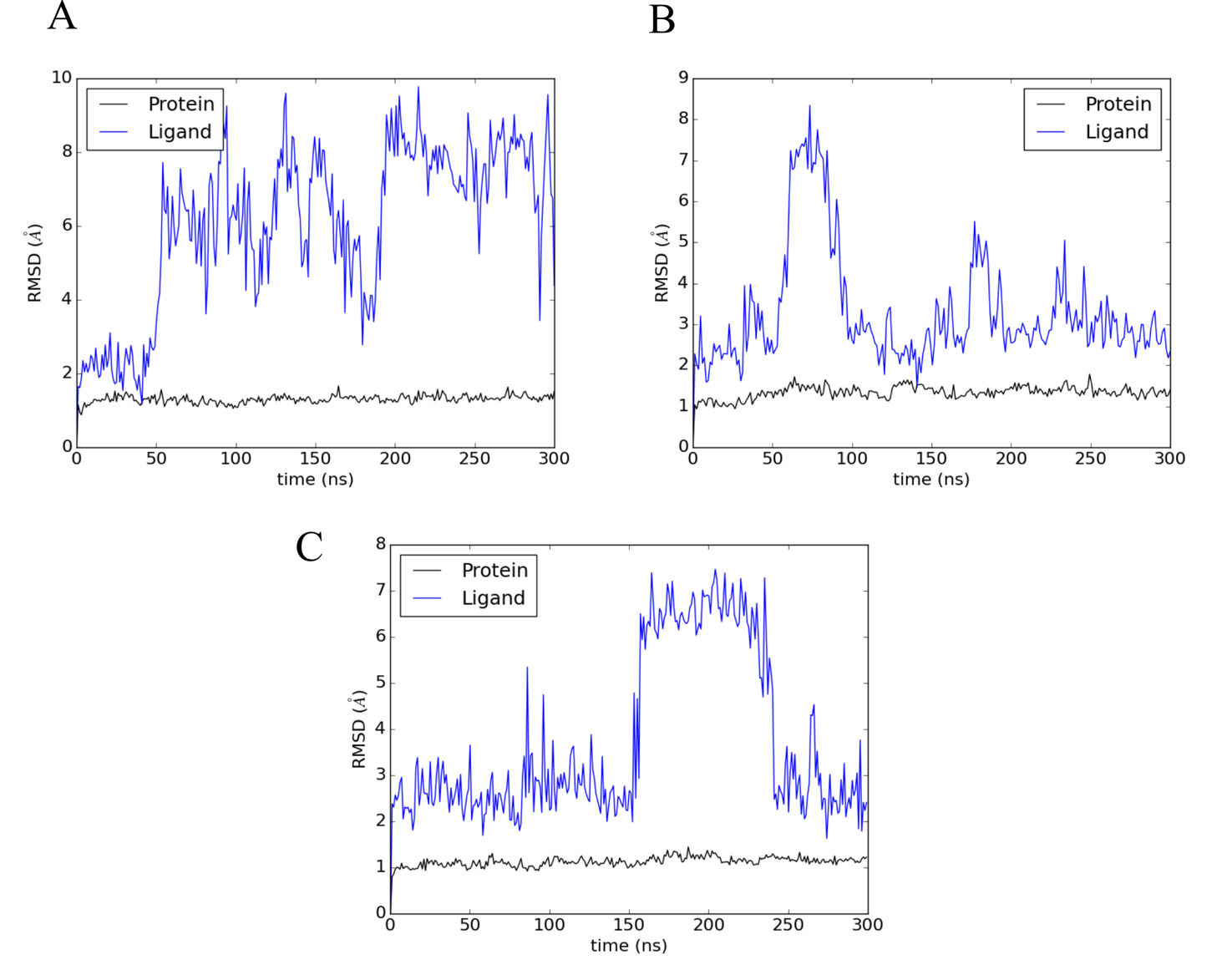


**Figure S1:** RMSD of protein backbone atoms and ligands during MD simulations of (A) CI, (B) CS pose 1, and (C) CS pose 7.


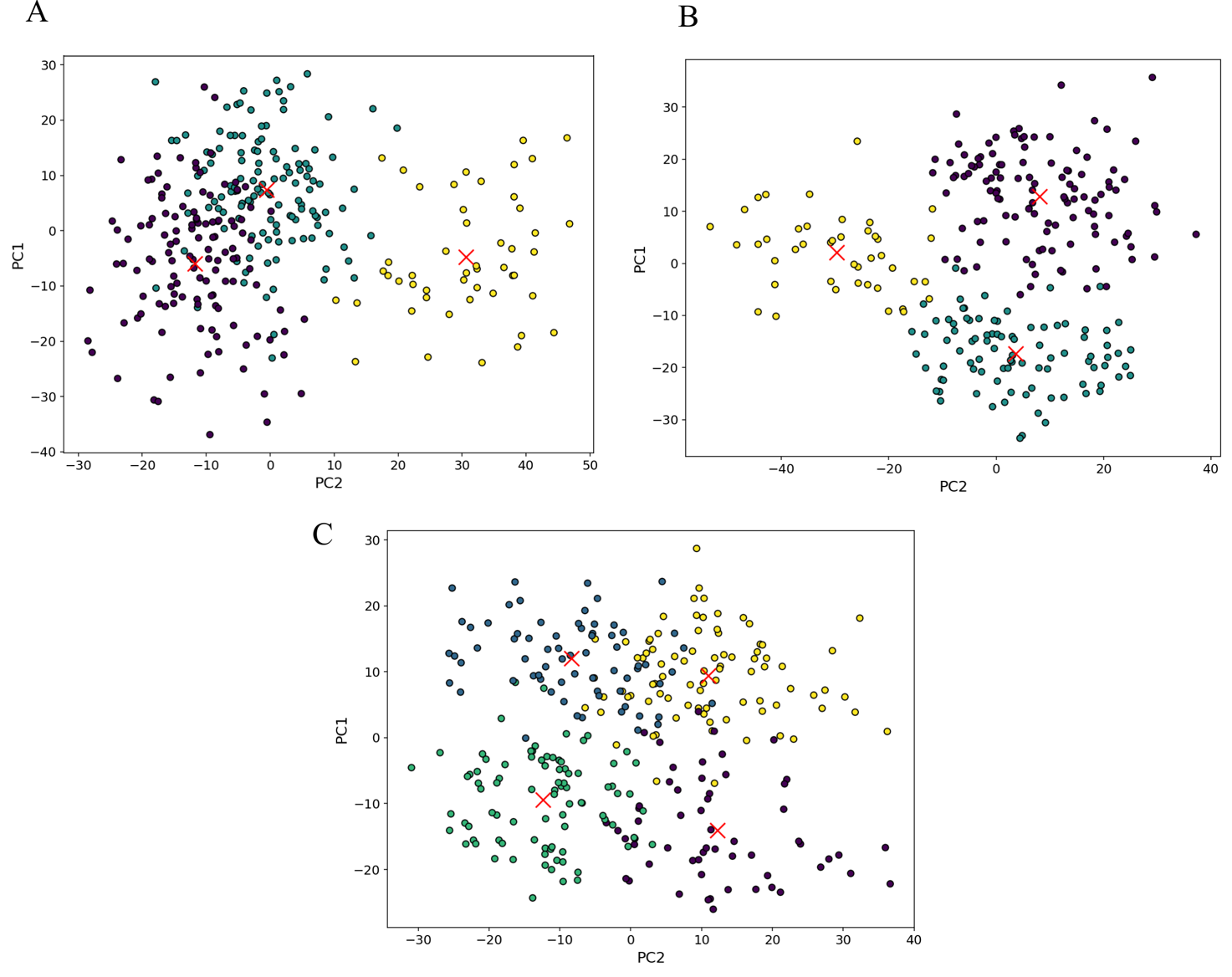


**Figure S2.** PCA plot showing the first and second principal components, with cluster labels obtained from the K-means++ algorithm applied to the features of (A) CI, (B) CS pose 1, and (C) CS pose 7 system

**1.** Selected physicochemical and ADME properties calculated using SwissADME.

| **Cpd** | **MW** | **Fraction Csp3** | **#Rotatable bonds** | **#H-bond acceptors** | **#H-bond donors** | **TPSA** | **LogP (Consensus)** | **LogS (ESOL)** | **ESOL Class** | **GI absorption** | **BBB permeant** | **Pgp substrate** | **Lipinski #violations** | **Bioavailability Score** |
| --- | --- | --- | --- | --- | --- | --- | --- | --- | --- | --- | --- | --- | --- | --- |
| **CI (Litreol)** | 318.49 | 0.62 | 13 | 2 | 2 | 40.46 | 5.96 | -5.89 | Moderately soluble | High | No | No | 1 | 0.55 |
| **CS** | 320.51 | 0.71 | 14 | 2 | 2 | 40.46 | 6.35 | -6.66 | Poorly soluble | High | No | No | 1 | 0.55 |
| **AS** | 404.58 | 0.68 | 18 | 4 | 0 | 52.60 | 7.01 | -7.15 | Poorly soluble | Low | No | No | 1 | 0.55 |
| **AI** | 402.57 | 0.60 | 17 | 4 | 0 | 52.60 | 6.73 | -6.61 | Poorly soluble | Low | No | No | 1 | 0.55 |


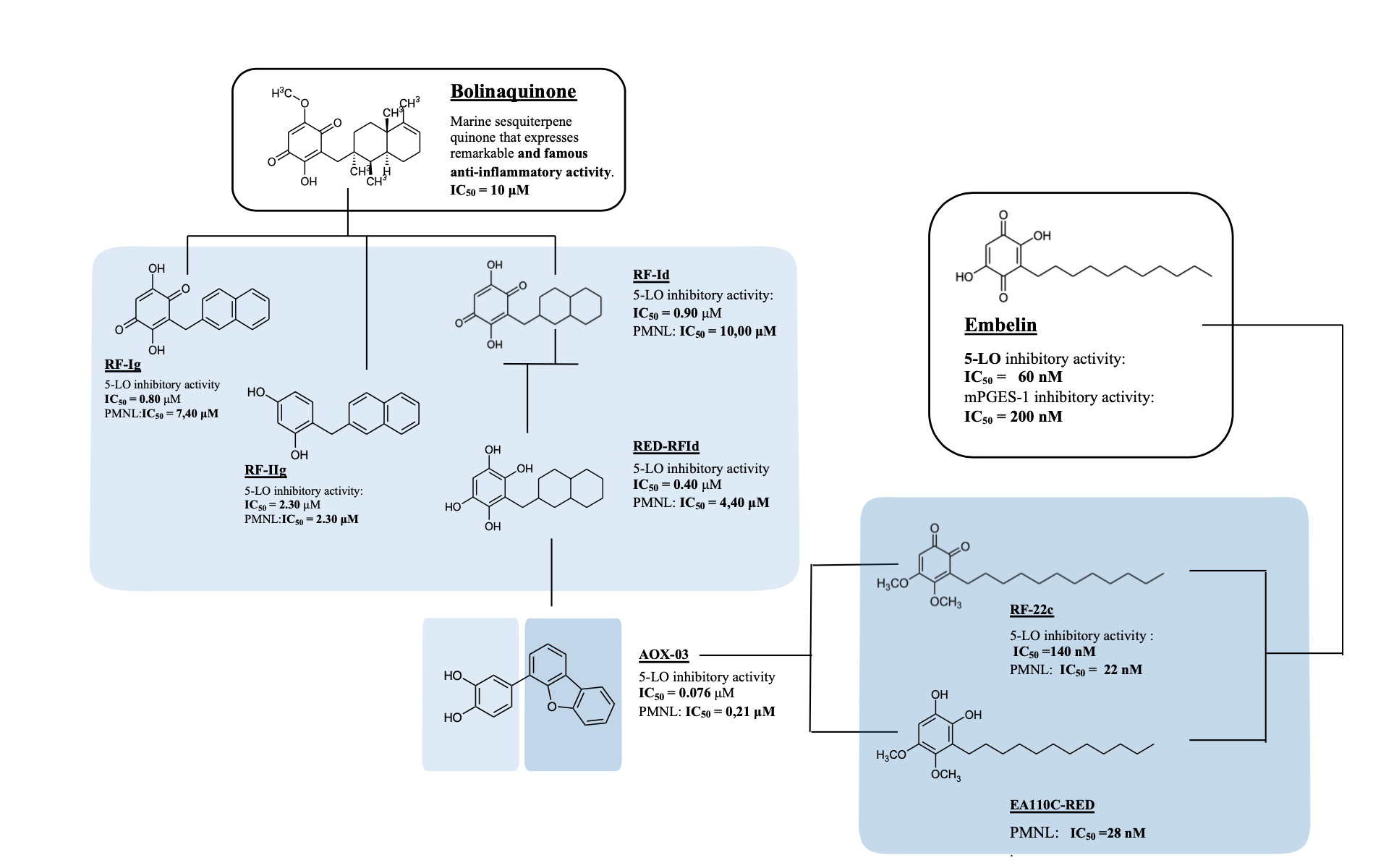


**Chart 1.** Structure optimization efforts in the target-focused approach, with rational optimization of the natural lead.
